# Viral vector platforms within the gene therapy landscape

**DOI:** 10.1038/s41392-021-00487-6

**Published:** 2021-02-08

**Authors:** Jote T. Bulcha, Yi Wang, Hong Ma, Phillip W. L. Tai, Guangping Gao

**Affiliations:** 1 Horae Gene Therapy Center, University of Massachusetts Medical School, Worcester, MA USA; 2 Department of Microbiology and Physiological Systems, University of Massachusetts Medical School, Worcester, MA USA; 3 Department of Pathophysiology, West China College of Basic medical sciences & Forensic Medicine, Sichuan University, Chengdu, China; 4 VIDE Program, University of Massachusetts Medical School, Worcester, MA USA; 5 Li Weibo Institute for Rare Diseases Research, University of Massachusetts Medical School, Worcester, MA USA

**Keywords:** Molecular medicine, Diseases

## Abstract

Throughout its 40-year history, the field of gene therapy has been marked by many transitions. It has seen great strides in combating human disease, has given hope to patients and families with limited treatment options, but has also been subject to many setbacks. Treatment of patients with this class of investigational drugs has resulted in severe adverse effects and, even in rare cases, death. At the heart of this dichotomous field are the viral-based vectors, the delivery vehicles that have allowed researchers and clinicians to develop powerful drug platforms, and have radically changed the face of medicine. Within the past 5 years, the gene therapy field has seen a wave of drugs based on viral vectors that have gained regulatory approval that come in a variety of designs and purposes. These modalities range from vector-based cancer therapies, to treating monogenic diseases with life-altering outcomes. At present, the three key vector strategies are based on adenoviruses, adeno-associated viruses, and lentiviruses. They have led the way in preclinical and clinical successes in the past two decades. However, despite these successes, many challenges still limit these approaches from attaining their full potential. To review the viral vector-based gene therapy landscape, we focus on these three highly regarded vector platforms and describe mechanisms of action and their roles in treating human disease.

## Introduction

**Fig. 1:**
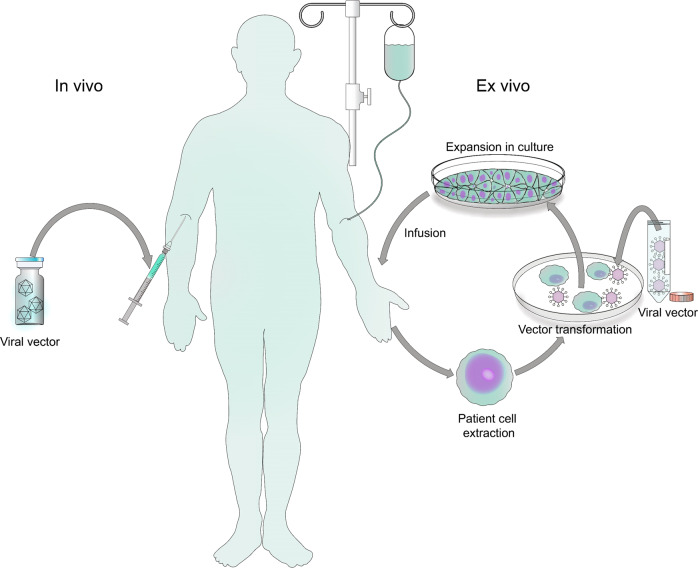
Summary of viral gene therapy modalities. In vivo gene therapy entails the direct administration of vector carrying a therapeutic transgene into the patient. Ex vivo gene therapy involves the extraction of a patient’s cells or from an allogenic source, genetic modification by a vector carrying a therapeutic transgene, selection and expansion in culture, and infusion to re-introduce the engineered cells back into the patient

Gene therapy is the treatment of a genetic disease by the introduction of specific cell function-altering genetic material into a patient. The key step in gene therapy is efficient gene delivery to the target tissue/cells, which is carried out by gene delivery vehicles called vectors. There are two types of vectors: viral and non-viral. Non-viral vectors will not be discussed in this review. Contemporary viral vector-based gene therapy is achieved by in vivo delivery of the therapeutic gene into the patient by vectors based on retroviruses, adenoviruses (Ads) or adeno-associated viruses (AAVs) (Fig. 1). Alternatively, a therapeutic transgene can be delivered ex vivo, whereby cells of a patient are extracted and cultured outside of the body. Cells are then genetically modified by introduction of a therapeutic transgene and are then re-introduced back into the patient. There are four basic gene therapy approaches as follows: gene replacement, the delivery of a functional gene to replace a non-working gene; gene silencing, inactivation of a mutated gene that has become toxic to cells; gene addition, over expression of a “foreign” or exogenous gene to impact cellular function; and gene editing, a permanent manipulation of a gene in a patient’s genome.

The first gene therapy trial, at least conceptually, was performed by Dr. Stanfield Rogers, who treated two sisters who had hyperargininemia.^1^ The treatment was based on his observations that patients with Shope papilloma virus had decreased serum arginine levels. Unfortunately, the trial failed to reverse the disease, as the Shope papilloma genome did not encode for arginase production. In a 1980 study that was not formally published, Dr. Martin Cline attempted to insert recombinant β-globin, a factor needed in the production of hemoglobin, into bone marrow cells of two patients with β-thalassemia.^2^ The transfected cells were then re-introduced back into the patients. Although groundbreaking at the time, this first attempt at an ex vivo gene therapy was ultimately a failure. Nonetheless, these two efforts were among several others that endeavored to deliver genetic material to patients, in the hopes of treating disease. It was not until the early 90s that viral vector gene therapies found clinical success. A trial led by French Anderson, Michael Blaese, and Steven Rosenburg also employed an ex vivo strategy in treating a patient named Ashanthi DeSilva, who had adenosine deaminase deficiency severe combined immunodeficiency disease (ADA-SCID). Several infusions of T cells transformed by a recombinant retrovirus carrying the ADA gene were administered, resulting in what is regarded as the first successful gene therapy in humans.^3,4^

**Fig. 2:**
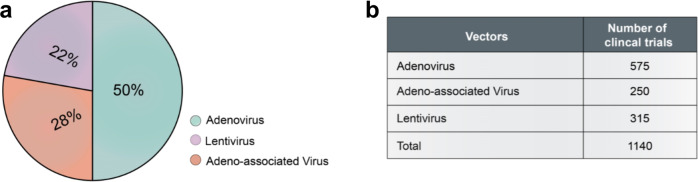
Summary of viral vectors used in clinical trials. **a** Pie chart showing the percentage of adenovirus, adeno-associated virus, or lentivirus vectors in use. **b** A table of the current number of clinical trials employing the different viral vectors. Data source: Wiley database on Gene Therapy Trials Worldwide. http://www.abedia.com/wiley/vectors.php

The use of viruses for therapy has long been practiced and actually belongs to a class of viral-based treatments known as virotherapies. Perhaps, the main reason why earlier viral-based therapies failed to achieve efficacy was due to a lack of full understanding of the viral biology. Now with 40 years of accumulated knowledge on viruses, promising viral vector-based strategies to treat genetic diseases are numerous. Some human diseases even have several effective treatment options to choose from. Typically, a viral vector is defined by three components as follows: (1) the protein capsid and/or envelope that encapsidates the genetic payload, and defines the vector’s tissue or cell tropism and antigen recognition; (2) the transgene of interest, which when expressed in cells, serves to confer a desired effect; and (3) the “regulatory cassette,” the combined enhancer/promoter/auxiliary elements that controls stable or transient somatic expression of the transgene as an episome or as a chromosomal integrant. Design aspects for these three components are different for each viral vector platform, have unique considerations, and harbor their own strengths and weaknesses. In this review, we will describe three viral vector platforms that have gained wide use for efficacious gene therapy and regulatory approval. These three strategies are based on Ads, AAVs, and lentiviruses (retroviruses), the viruses that a majority of gene therapy vectors are based upon (Fig. 2). For each of these vector platforms, we will review their general compositions and their mode of infection, highlight key vector platforms and their biological properties, describe current production strategies in use, feature their uses as commercialized drugs and in clinical trials, and finally discuss challenges and future outlooks.

### Ad vectors: large cargo capacities for transient targeted gene delivery

#### Structure and genome

**Fig. 3:**
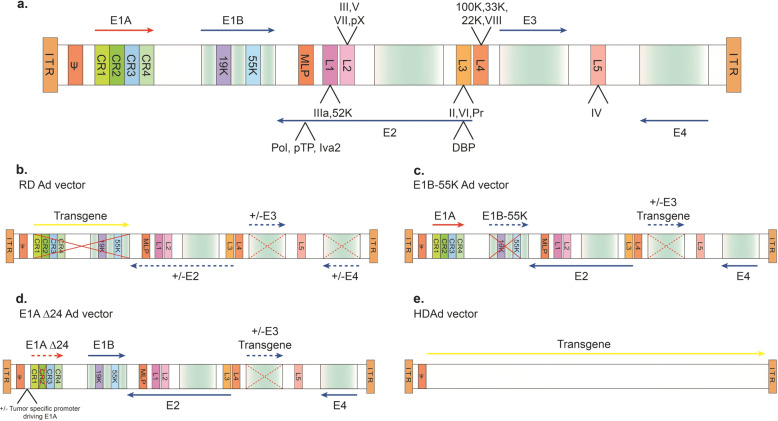
Schematic of the wild-type adenovirus type 5 (Ad5) genome and the genetic modifications of common Ad5-based vectors. **a** Diagram of the wild-type Ad5 genome structure. The 36 kb genome consists of four early transcription elements (E1, E2, E3, and E4), five late expression genes (L1–L5), *cis*-packaging elements (ψ) and two inverted terminal repeat sequences (ITR). The E1A (red arrow) gene contains four conserved domains (CR1-4), each of which interacts with special cellular proteins. The E1B gene encodes for two distinct tumor antigens, the 19 kDa (19K) and 55 kDa (55K) proteins. The E2 gene encodes DNA-binding protein (DBP), terminal protein (pTP), IVa2, and DNA polymerase (Pol). The E4 gene encodes 1–7 open reading frames. The major late messenger RNAs (L1–L5) mainly encode for virion structural proteins and are derived from a pre-mRNA, which is driven by a major late promoter (MLP) via alternative splicing and polyadenylation. L1 encodes for IIIa and 52K. L2 encodes for the penton base gene (capsid protein III) and the core proteins V, pVII, and pX. L3 encodes for the hexon (capsid protein II), capsid protein precursor (pVI), and protease (Pr) genes. L4 encodes for 100K, 33K, 22K, and pVIII. L5 encodes for the fiber gene (capsid protein IV). **b**–**e** Diagrams of rAd vectors. **b** Replication-defective (RD) vector. The E1A and E1B regions are deleted and replaced with an expression cassette containing an exogenous promoter and a transgene of interest (indicated by the solid red X and yellow arrow). The E3 and E4 regions are usually deleted to accommodate larger insertions and eliminates leaky expression of other viral genes. **c** E1B-55K replication-competent vector. The E1B-55K region is deleted (solid red X and dashed blue arrow), whereas in some vectors, the E3 region is deleted and replaced with an expression cassette (dashed red X and dashed blue arrow). **d** E1A-Δ24 (Δ24) replication-competent vector. The CR2 region in E1A is deleted (solid red X and dashed red arrow) and the E1A promoter can be replaced with various tumor-specific promoters to drive CR2-deleted E1A expression. In some vector designs, the E3 region is deleted and replaced with an expression cassette (dashed red X and dashed blue arrow). **e** Helper-dependent Ad vectors (HDAds). Most or all of the Ad genomic elements are replaced with a therapeutic expressing cassette (yellow arrow), save for the *cis*-packaging sequences (ψ) and ITRs. These vectors are propagated in the presence of an Ad helper vector

Ad is a non-enveloped virus that is known to mostly cause infections of the upper respiratory tract but can also infect other organs such as the brain and bladder. It possesses an icosahedral protein capsid that accommodates a 26- to 45-kb linear, double-stranded DNA genome. The Ad genome is flanked by hairpin-like inverted terminal repeats (ITRs) that vary in length (30–371 bp at its termini).^5,6^ The ITRs serve as self-priming structures that promote primase-independent DNA replication. A packaging signal located at the left arm of the genome is required for viral genome packaging. The Ad genome encodes ~35 proteins that are expressed in the early and late phases of viral gene transcription. The Ad genome comprises five so-called “early-phase” genes, *E1A*, *E1B*, *E2*, *E3*, and *E4*.^7^ The early-phase genes are transcribed before the initiation of viral DNA replication (about 7 h post infection). The “immediate-early” *E1A* gene is essential for transcription of other viral genes (e.g., *E1B*, *E2*, *E3*, and *E4*), which are responsible for viral DNA synthesis and play roles in modulating expression of host genes. E1B plays roles in counteracting the cell’s activation of apoptosis by binding and inactivating p53, permitting viral replication to progress.^8^ The “late-phase” genes (L1–L5) are generally required for virus assembly, release, and lysis of the host cell.^9^ These gene products are derived from the five late transcriptional units that are produced by alternative splicing and polyadenylation of the major late messenger RNAs (Fig. 3a).^10^

The Ad viral capsid is 90–100 nm in diameter. It is composed of the structural proteins, hexon (capsid protein II), penton base (capsid protein III), fiber (capsid protein IV), capsid protein precursors pIIIa, pVI, and pVIII, and capsid protein IX, and the virion core proteins (V, VII, and X).^11^ Hexons are the most abundant structural components residing on the surface of the virus, comprising 240 trimers in the assembled capsid. There are 12 penton proteins located at the apex of the icosahedral vertices, giving rise to the protruding fibers. The V, VII, and X proteins mainly associate with the viral genome and play critical roles in genome replication, condensation, and assembly processes.^11^ The IIIa proteins are located at the inner surface of capsid, and drive the assembly of the packaged genome via binding with L1 52/55K and stabilization of the vertex regions via interaction with penton base, hexon, and VI proteins.^12,13^ The VI proteins link the core to the inner icosahedral shell, while also serving as lytic factors during endosomal disruption.^14^ The VIII proteins bind to the peripentonal hexons to stabilize the viral capsid.^15^ The terminal protein (pTP) covalently links to the 5′-ends of the Ad genome and enhances genome replication.^16^

Ad assembly is thought to proceed by the following sequential pathway steps: (1) empty capsids (procapsids) are assembled with capsomers (hexons, penton bases, and fibers) along with some minor capsid proteins and unstructured proteins; (2) the viral genome binds to packaging proteins (IVa2, L4 33K, L1 52/55K, and L4 22K) via the packaging signal (ψ) within the left ITR; (3) encapsidation of the viral genome into the procapsid through a hypothetical portal located at a unique vertex that is accompanied by the release of scaffolding and some of the packaging proteins; and (4) cleavage of the precursor proteins (pIIIa, L1 52/55K, pVI, pVII, pVIII, mu, and pTP) by the adenovirus protease (AVP), resulting in the final mature viral particle. Two to 3 days after entering the cell nucleus, new virions are assembled and cells lyse to release virions.^17^

#### Infection pathway

To date, more than a hundred human Ad genotypes have been identified, falling into seven subgroups classified A to G (http://hadvwg.gmu.edu). Knowledge on the Ad infection pathway is largely based on human Ad5 (HAd5). Infection initiates with interaction between the cell surface-localized coxsackievirus-Ad receptor (CAR) and the distal domain of the virus capsid fiber.^18,19^ In addition, many other receptors for entry of Ads have also been found, such as CD46, DSG2, and sialic acid.^20–22^ Binding of the virus to the cell surface is then followed by endocytosis, which is mediated by interaction between the tripeptide Arg-Gly-Asp (RGD) motif in the penton base and the αV integrins on the host cell surface.^23^ Following endocytosis, the capsid is disassembled and the V and VI proteins facilitate endosomal escape.^14,24^ The viral DNA subsequently enters the nucleus through the nuclear envelope pore complex.^25^ In addition, hexons from partially disrupted virions bind with dynein motors to help the virus traffic to the nuclear pore via the microtubular network.^26^ In the nucleus, the viral DNA predominantly remains epichromosomal and is not incorporated into the host cell genome.^27^

#### Ad natural infections and pathological association

Most people carry neutralizing antibodies (NAbs) against one or more of the prevalent human Ad serotypes, as a result of exposure to Ads from past infections.^28,29^ Ads typically result in asymptomatic responses or lead to mild or severe disease in immunocompetent individuals.^30^ Among the seven known species of human Ads, species B and C are usually acquired in early childhood and cause infections in the upper respiratory, gastrointestinal, and urinary tracts. Some serotypes in species D cause epidemic keratoconjunctivitis, whereas HAd4 from species E causes acute respiratory disease.^31^ Due in part to the epidemiology of Ads in the human population and Ad antigen-specific T cells, which result in the lifelong immunity, vectors based on Ad tend to have compromised potencies^32,33^ and trigger a stronger immunological response compared to other viral vectors, such as those based on AAV (discussed further below).

#### Ad as a vector in gene therapy

Ad vectors have the following advantages: (1) high transduction efficiency, both in quiescent and dividing cells; (2) epichromosomal persistence in the host cell; (3) broad tropism for different tissue targets; and (4) and the availability of scalable production systems.^34^ Contemporary Ad vectors are derived from human serotypes HAd2 and HAd5. The major objectives in Ad vector development are to overcome the challenges associated with its widely pre-existing viral immunity among the general population, life-threatening strong innate immune responses to its capsid proteins, and robust adaptive immune responses to de novo synthesized viral and transgene products.^35^ Since the first generation of E1A-deleted Ad vectors were established, various strategies have been developed to improve their capacity, efficacy, gene transfer longevity, and safety.

*First generation*. The first generation of Ad vectors were engineered by replacing the E1A/E1B region with transgene cassettes that can be up to 4.5 kb in length (Fig. 3b). Removal of the *E1A* gene results in the inability of recombinant Ad (rAd) to replicate in the host cell.^36^ Therefore, complementary cell lines designed to express E1A and E1B, such as HEK293, are needed for production.^37^ As the E3 region is not required for viral propagation in cultured cells, the E1/E3 double-deletion frees up more space for the transgene cassettes (6.5 kb in length). The first generation of Ad vectors has two main disadvantages as follows: (1) de novo expression of Ad proteins can still activate the host immune response, causing clearance of vector-transduced cells;^38^ and (2) possible spontaneous homologous recombination between the vector and engineered E1 region from HEK293 during genome amplification can generate replication-competent adenovirus.

*Second generation*. Due to issues with first-generation Ad vectors, researchers developed improved versions by further deleting the other early gene regions (E2a, E2b, or E4), permitting additional space for larger transgene cassettes (10.5 kb) (Fig. 3b). These new vector designs include temperature-sensitive rAd vectors, generated by ablation of E2A-encoded DNA-binding protein,^39,40^ deletion of the E2b-encoded DNA polymerase (Pol) protein,^41,42^ and deletion of the E4 region.^43^ All of these Ad vectors were shown to have significantly decreased late gene expression and elicit less of a cytotoxic T-lymphocyte response when administered in vivo. As a result, transgene expression was substantially prolonged in mice compared to first-generation vectors.^39–43^ Again, the deleted genes are complemented by engineered production cell lines. Unfortunately, the deletion of E2 and/or E4 genes negatively affects viral vector amplification, resulting in lower titers.^44^ This reduction is a consequence of inefficient complementation of E2/4 with engineered cell lines. Despite the changes, the native Ad late genes that are still retained within the vector genome can trigger host immunogenicity and cellular toxicity.^45^

*Third generation*. Third-generation Ad vectors, referred to as “gutless” or “helper-dependent” Ad vectors, have all viral sequences deleted, except for the ITRs and the packaging signal (Fig. 3e). These vectors, also called “high-capacity” adenoviral vectors (HCAds),^46^ can accommodate ~36 kb of space for cargo gene(s). Production of HCAds in cell culture requires an additional adenoviral helper virus (HV) that is similar in composition to first-generation vectors, but with the distinction that they contain loxP sites inserted to flank the packaging signal. The HCAd genome is transfected into HEK293 producer cells that express Cre recombinase, along with the HV infection. Replication and packaging are permitted by the viral proteins provided by the helper genome. The engineered Cre in producer cells ensures that only the HCAd genome can be packaged, as the helper-virus genome-packaging signal is excised by Cre-mediated recombination at the loxP sites.^47^ Compared with the previous generations of Ad vectors, HCAds have reduced immunogenicity, prolonged transduction in the host cell, and a significantly larger cargo capacity, which can accommodate multiple transgene cassettes, or therapeutic genes that are driven by their larger native promoters and enhancers to mimic physiological levels of expression. However, the main challenge in HCAd production is ensuring that the helper adenovirus is eliminated from vector preparations, which may alter efficacy and safety of HCAd vectors in vivo.

*Conditionally replicating Ad vectors*. The engineering of tumor-specific gene promoters into the Ad genome can be used to control the initiation of viral replication to create conditionally replicative adenoviral vectors (CRAds). The first CRAds were based on the partial deletion of the E1B sequence (E1B-55K) (Fig. 3c).^48^ As viral genome replication can be completed without E1B-55K in most tumor cells, as they lack p53, E1B-55K-deleted Ads only undergo genome amplification and subsequent lysis in a patient’s tumor cells, leaving non-tumor cells unaffected. In the next generation of CRAds, the 24-amino acid CR2 domain of E1A protein was deleted to generate AdD24 vectors (Fig. 3d).^49^ The CR2 domain is known to bind retinoblastoma protein to release E2F, the S-phase-activating transcription factor required for viral genome replication in normal cells. As cancer cells express excessive amount of E2F, Ad replication can proceed without E1A. The resulting dl922-947 and AdD24 vectors both showed high replication potency and selectivity in tumor cells.^50^ These oncolytic Ad vectors are unique from other natural oncolytic viruses such as reoviruses, senecaviruses, and the α-virus M1 virus.^51,52^ Improvements to tumor specificity through these engineering efforts have inherent advantages over natural viruses, as natural viruses carry wild-type viral genomes that may result in unknown consequences.

#### Therapeutic Ad vectors and commercial availability

In the early 1990s, Ad vectors for in vivo therapy were first used to deliver and express the α-1 antitrypsin (*A1AT*) gene in rat hepatocytes and lung tissues.^53,54^ Following this demonstration, many Ad-based gene delivery trials for treating human monogenic disease were conducted, including the expression of cystic fibrosis transmembrane conductance regulator (*CFTR*) in lung tissues of patients with cystic fibrosis, and vascular endothelial growth factor (VEGF) in patients with peripheral vascular disease.^55,56^ Unfortunately, a series of studies revealed that Ad is strongly immunogenic, which not only restricted the delivery and expression of exogenous genes but also caused undesired immune responses in treated subjects.^57^ Soon after in 1999, the death of Jesse Gelsinger, who was enrolled in a clinical trial for *OTC* (ornithine transcarbamylase) gene therapy with an Ad vector,^58^ raised safety concerns for human gene therapies and caused a significant decline in related studies for the following decade. It was discovered that innate response to the capsid protein triggered cytokine storm.^58^ These unfortunate results and backup evidence in animal models indicated that even with gutless rAd designs, the capsid may still trigger a strong immune response towards the therapy,^59,60^ thus limiting the use of Ad vectors in human gene therapy.

It is now well-accepted that Ad can trigger strong immune responses in humans, reinforcing safety concerns for their application.^61^ However, for therapies that are not impacted by an immunological response, but may even rely on them to kill the cells they transduce, Ad vectors have seen significant utility. For example in 2003, Gendicine, an Ad vector harboring a Rous sarcoma virus promoter-driven p53 gene, was approved in China as the world’s first commercialized gene therapy drug for cancer.^62^ ONYX-105 (dl1520) was the first CRAd to enter clinical trials,^63,64^ followed by a similar replication-selective Ad vector called H101 (Oncorine), which also gained commercial approval in China.^65^ Although numerous clinical results have confirmed the safety of ONYX-105 and H101, the drugs were ultimately not very effective. The major reason for the lowered efficacies is that deletion of E1B-55K also causes attenuated viral replication, even in tumor cells in vivo.

#### Ad vectors in clinical trials

Ad vectors have seen a rebirth in human gene therapy research. They maintain many practical advantages, including their broad tropism profiles, lack of host genome integration, and large packaging capacities (~36 kb). Currently, Ad-based gene therapy clinical trials account for 50% of total worldwide trials (Fig. 2a). They have been mainly applied towards novel vaccines and cancer therapies.

*Ad-mediated genetic vaccines*. Immunogenicity is a critical hurdle for viral vector efficacy, but has been exploited in the development of Ad-based vaccines.^66^ rAd vectors can deliver foreign epitopes to enhance the host immune response to pathogens by boosting the production of pro-inflammatory cytokines and effective adaptive humoral and cellular immune responses.^67^ These innovative approaches have made Ad vectors an ideal vaccine carrier.

For example, Ebola vaccines based on Ad vectors showed induction of specific antibody and T-cell responses in clinical trial subjects.^68–70^ Notably, HAd26-ZEBOV/MVA-BN-Filo (ClinicalTrials.gov identifier: NCT04028349) has been shown to be well-tolerated and produced durable humoral immune responses for more than a year post vaccination. A vector based on a non-human primate-derived Ad (ChAd3-ZEBOV) was also well-tolerated in both adults and children.^71,72^ In addition, human Ad-based influenza vaccines that can confer expression of major influenza viral antigens, such as HA, NP, and M2, have been tested in clinical trials including the H1N1 and H5N1 (NCT03232567 and NCT00755703).^73^ A chimpanzee Ad vector, ChAdOx1, expressing NP and M1 antigens were also designed and used in two phase I trials (NCT01818362 and NCT01623518).^74,75^ Ad-mediated human immunodeficiency virus (HIV) vaccines that individually confer expression of the HIV-1 genes *gal*, *pol*, and *env* (HAd5-gag, HAd5-pol, and HAd5-Nef, respectively) have also been developed and tested.^76–79^ However, these trials did not reveal efficacious vaccination. At the time of this review, no Ad-based HIV vaccine has been proven successful.

The global COVID-19 pandemic, which is caused by the severe acute respiratory syndrome coronavirus 2 (SARS-CoV-2), is currently the most threatening public health emergency. At the time that this review was written, SARS-CoV-2 has resulted in more than 100 million infection cases with more than 2 million deaths worldwide. In response, multiple vaccine strategies have been under development. An HAd5-based vaccine that delivers the SARS-CoV-2 spike glycoprotein was launched in a phase I clinical trial with 108 participants. Thus far, outcomes showed rapid humoral and T-cell responses after 14 days post vaccination in most participants, with no serious adverse events.^80^ A phase II clinical trial with a group of 508 participants to evaluate its efficacy has recently begun. In addition, a phase I/II clinical trial to evaluate the efficacy of a SARS-CoV-2 vaccine based on the chimp-derived ChAdOx1 capsid is also underway (NCT04324606).^81^ Preliminary data demonstrated that ChAdOx1-nCoV-19 treatment induced both humoral and cellular immune responses against SARS-CoV-2, with NAbs in 91% of participants after a single dose and 100% response after a booster dose.

rAds have also been used to deliver vaccines for cancer prevention. The overall strategy for immunizing against tumor cells is to induce the expression of tumor-associated antigens (TAA) and/or oncolytic processes that promote antitumor immune responses via Ad-mediated gene delivery. Currently tested antigens include prostate-specific antigen for prostate cancer (NCT00583752 and NCT00583024), MAGE-A3 for solid tumor, human papilloma virus (HPV) E6/E7 for HPV-associated cancers (NCT02285816, NCT02879760, and NCT03773744), and carcinoembryonic antigen (CEA) for colorectal and pancreatic cancers (NCT03329248 and NCT03387098). A non-human-derived AdOx1 was employed to deliver the oncofetal TAA, 5T4, to immunize against prostate cancers (NCT03815942 and NCT02390063). Using HAd5 vectors to express a combination of TAAs, such as CEA + brachyury + MUC1 and CEA + brachyury + mucin 1 (MUC1) + Her2 are also used to promote cancer vaccination.^82^

*Anticancer therapy*. To date, various approaches using Ad vectors to specifically kill tumor cells have been developed and tested in clinical trials (Table 1). The early generation of Ad vector-based anticancer therapies mainly relied on replication-defective vectors for their immunogenic properties to deliver tumor repressor, cytotoxic, or immune-regulating genes to induce tumor cell death and antitumor immune responses.

**Table 1:** A selection of ongoing and completed clinical trials employing Ad vectors

Conditions	Intervention	Transgene	Trial stage	Identifier
Prostate cancer	CG7870 combination with Docetaxel	E1A under control of the rat probasin promoter and E1B under control of the PSA promoter-enhancer	Phase I/II	NCT00103428
Prostate cancer	CG7870	E1A under control of the rat probasin promoter and E1B under control of the PSA promoter-enhancer	Phase I/II	NCT00116155
Metastatic melanoma	OBP-301 (Telomelysin)	E1A/E1B under control of human telomerase reverse transcriptase gene hTERT promoter	Phase II	NCT03190824
Esophagogastric adenocarcinoma	OBP-301 (Telomelysin)	E1A/E1B under control of human telomerase reverse -transcriptase gene hTERT promoter	Phase II	NCT03921021
Solid tumors	Ad/PNP coupled with fludarabine phosphate	PNP	Phase I	NCT01310179
Hormone-refractory metastatic prostate cancer	Ad-OC-TK + valacyclovir	HSV-TK gene under control of osteocalcin promoter in the area of the excised E1 region	Phase I/II	in Japan
Prostate cancer	AdV/RSC-TK + Brachytherapy	HSV-TK gene under control of Rous sarcoma virus long terminal repeat promoter RSV	Phase II	NCT01913106
Prostate cancer	CTL102 + CB1954	NTR under control of CMV promoter	Phase I/II	40 Patient cohort in UK
Advanced pancreatic cancer	Ad5-yCD/mutTK(SR39)rep-ADP	CD/TK/ADP	Phase I	NCT02894944
Prostate cancer	Ad5-yCD/mutTK(SR39)rep-ADP + IMRT intensity-modulated radiation therapy	CD/TK/ADP	Phase I/II	NCT00583492
Prostate cancer	Ad5-yCD/mutTK(SR39)rep-hIL12	CD/TK/hIL12	Phase I	NCT02555397
Malignant pleural mesothelioma	rAd-IFN administered with Celecoxib and Gemcitabine	IFN-α-2b	Phase III	NCT03710876
Advanced peritoneal malignancies	Oncos-102 + Durvalumab	GM-CSF	Phase I/II	NCT02963831
Metastatic castration-resistant prostate cancer	Oncos-102 + DCVAC/PCA	GM-CSF	Phase I/II	NCT03514836
High-grade NMIBC after BCG failure	CG0070	E1A under control of E2F-1 promoter, CM-CSF under control of E3 promoter	Phase II	NCT02365818
Mutiple advanced cancer	LOAd703	CD40L and 4-1BBL under control of CMV promoter	Phase I/II	NCT03225989
Pancreatic cancer	LOAd703	CD40L and 4-1BBL under control of CMV promoter	Phase I/II	NCT02705196
Malignant melanoma	LOAd703 + Atezolizumab	CD40L and 4-1BBL under control of CMV promoter	Phase I/II	NCT04123470
Recurrent glioblastoma	DNX-2401	NA	Phase I	NCT00805376
Recurrent glioblastoma	DNX-2401 + Temozolomide	NA	Phase I	NCT01956734
Recurrent glioblastoma or gliosarcoma brain tumors	DNX-2401 + IFN-γ	NA	Phase I	NCT02197169
Naive diffuse intrinsic pontine gliomas	DNX-2401	NA	Phase I	NCT03178032
Recurrent high-grade glioma	DNX-2401	NA	Phase I	NCT03896568
Advanced or metastatic melanoma	ICOVIR-5 (Ad-DM-E2F-K-Delta-24-RGD)	E1A under control of E2F-1 promoter and myotonic dystrophy locus insulator DM-1	Phase I	NCT01864759
Refractory solid tumors	ICOVIR-7	E1A under control of E2F-1 promoter with four new E2F-responsive palindromes		21 Patient cohort in Finland
Advanced pancreatic cancer	VCN-01 + Gemcitabine and Abraxane®	E1A under control of E2F-1 promoter and myotonic dystrophy locus insulator DM-1, and Hyaluronidase	Phase I	NCT02045589
R/M head and neck squamous cell carcinoma	VCN-01 + Durvalumab	E1A under control of E2F-1 promoter and myotonic dystrophy locus insulator DM-1, and Hyaluronidase	Phase I	NCT03799744
Refractory retinoblastoma (RTB)	VCN-01	E1A under control of E2F-1 promoter and myotonic dystrophy locus insulator DM-1, and Hyaluronidase	Phase I	NCT03284268
Resectable colon cancer, non-small cell lung cancer, bladder cancer, renal cell carcinoma	ColoAd1 (Enadenotucirev)	NA	Phase I	NCT02053220
Locally advanced rectal cancer	Chemoradiation + Enadenotucirev	NA	Phase I	NCT03916510
Metastatic cancer, epithelial tumor	NG-641	FAP-TAc antibody /CXCL9/CXCL10/IFN-α	Phase I	NCT04053283
Metastatic cancer, epithelial tumor	NG-350A	anti-CD40 antibody	Phase I	NCT03852511

*CD* cytosine deaminase, *CD40L* CD40 ligand, *CMV* cytomegalovirus, *CXCL* chemokine ligand, *FAP-Tac* fibroblast-activating protein/CD3, *GM-CSF* granulocyte-macrophage colony-stimulating factor, *hIL12* human interleukin-12, *HSV-TK* herpes simplex virus thymidine kinase, *hTERT* *human telomerase reverse transcriptase*, *IFN* interferon, *NA* not applicable, *NTR* *Escherichia coli* nfsB bacterial nitroreductase, *PNP* *Escherichia coli* Pruine nucleoside phosphorylase, *PSA* prostate-specific antigen.

i. Delivery of suicide genes. Based on the well-established fact that many tumor types display dysfunction in the p53 tumor repressor pathway,^83^ Ad vectors have been engineered to induce p53 expression to cause cell-cycle arrest and apoptosis in tumor cells.^84,85^ However, not all tumor types lack p53 function. Other applications for Ad vectors in anticancer therapy have been tested in the targeted expression of pro-drug-converting enzymes to achieve tumor cell killing. For example, the enzyme purine nucleoside phosphorylase (PNP) converts the pro-drug fludarabine monophosphate (F-ara-AMP) into fluoroadenine, which confers cytotoxicity to proliferating cells. A dose-escalation phase I trial to treat patients with advanced tumors was conducted to test the efficacy of an Ad vector encoding *Escherichia coli* PNP (Ad/PNP), followed by intravenous administration of F-araAMP (NCT01310179).^86^

The herpes simplex virus thymidine kinase (HSV-TK) can convert the pro-drug ganciclovir (GCV) to a cytotoxic nucleotide to selectively kill dividing cells. In 2004, the first randomized, controlled clinical trial with the non-replicable Ad HSV-TK/GCV (AdHSV-TK/GCV) gene therapy was carried out (NCT00005025).^87^ Since then, Ad-TK vectors have been applied in multiple clinical trials around the world for treating numerous types of solid tumor cancers. In 2006, a phase I/II clinical trial was conducted with an Ad-TK vector under the control of the Rous sarcoma virus long terminal repeat (LTR) promoter (Ad-RSV-TK) to treat prostate cancer (NCT01913106).^88^ Another phase I/II trial used an Ad vector to express TK with the bone-specific osteocalcin promoter to treat patients with hormone-refractory prostate cancer exhibiting metastasis to the bone.^89^

Cytosine deaminase (CD) can convert the pro-drug 5-fluorocytosine (5-FC) to toxic 5-fluorouridine (5-FU), which is further processed in cells to 5-FUTP and 5-FdUMP. These products cause a blockage of thymidylate synthase and subsequent damage to DNA. The HAd5-CD/TKrep vector, which contains the CD/HSV-TK chimeric enzyme transgene, showed long-term effectiveness when systemically delivered along with 5-FC and GCV, and was used in conjunction with intensity-modulated radiotherapy (IMRT) (NCT00583492).^90^ A modified version of this treatment (HAd5-yCD/mutTK(SR39)rep-ADP), which delivers a yeast CD fused to a mutated TK enzyme and the Ad death protein (ADP) showed promise in a phase I clinical trial for advanced pancreatic cancer (NCT02894944).^91^ A variant of this vector, HAd5-yCD/mutTK(SR39)rep-hNIS, also co-delivers a transgene encoding the human sodium iodide transporter (hNIS) to enable imaging of tumors to monitor viral spread and efficacy by single photon radionuclide computed tomography (SPRCT) and positron emission tomography.^92^ Another vector that was designed by the same research group also harbors the pro-inflammatory cytokine IL-12 to induce an antitumor immune response.^93^ Currently, two clinical trials using this strategy to treat prostate and pancreatic cancer are ongoing (NCT02555397 and NCT03281382, respectively).

ii. Delivery of immuno-regulatory genes. Ad vectors can also be armed with immune-regulating genes to stimulate antitumor immune responses in the patient. Intrapleural administration of Ad-delivered interferon (IFN)-β or IFN-α-2b to the lungs have been proven to be safe treatments for malignant pleural mesothelioma.^94,95^ Most recently, treatments using Ad-IFN-α-2b in combination with celecoxib and gemcitabine are being actively tested in a phase III trial (NCT03710876).

Granulocyte-macrophage colony-stimulating factor (GM-CSF) is known to induce activation of immune cells to trigger an antitumor response. Replication-competent HAd5/3 chimeric viruses expressing GM-CSF (Oncos-102, HAd5/3-D24-GM-CSF) were tested in a phase I clinical trial to target solid tumors.^96,97^ Since then, Oncos-102 has been tested in a phase I clinical trial, and in combination with durvalumab and autologous dendritic cell immunotherapy (DCVAC/PCa) in phase I/II trials for treating solid tumors (NCT01598129, NCT02963831, and NCT03514836). CG0070 is also a replication-competent oncolytic Ad vector that expresses GM-CSF under control of the human E2F-1 promoter to target bladder cancer. The treatment underwent a phase II clinical trial (NCT02365818).^98^ The interim results demonstrate that CG0070 confers acceptable levels of toxicity and an overall 47% complete response rate after six months for all patients and 50% for patients with carcinoma in situ.^99^

CD40 is a cell surface receptor that has also been shown to prevent cell proliferation and promote apoptosis once it interacts with CD40 ligand (CD40L). This association stimulates T-helper 1 immunity via maturation of dendritic cells and promotion of M2 to M1 macrophage differentiation.^100^ In most breast cancer cells, CD40 is overexpressed, permitting the possibility of using Ad vectors to deliver CD40L to target breast cancers. A vector that expresses CD40L under the control of a promoter containing a hypoxia-response element and an estrogen response element (AdEHCD40L) showed cancer-specific killing ability.^101^ Another Ad vector expressing CD40L under the hTERT promoter (CGCT-401) was developed to inhibit tumor growth via oncolysis and apoptosis.^102^ LOAd703 is an immuno-stimulatory oncolytic virus developed by the same research group and uses an HAd5/35 vector to express trimerized CD40L and 4-1BBL, another peptide known to enhance immunologic memory and expand natural killer cells to stimulate innate and adaptive immune responses.^103^ LOAd703 is currently being tested in phase I/II trials for pancreatic cancer patients (NCT03225989, NCT02705196, and NCT04123470).^104^

iii. Chimeric and tropism-modified oncolytic Ad vectors. Low expression levels of CARs, the primary Ad receptor on tumor cells, often results in resistance to infection by CAR-dependent oncolytic Ad vectors. To further enhance the cancer-selectivity of Ad vectors, researchers have sought alternative Ad-receptor interactions. Delta-24-RGD (DNX-2401) is an oncolytic Ad vector containing a deletion of the conserved region 2 of E1A (E1AΔCR2) and an insertion of an ACDCRGDCFCG peptide sequence (RGD-4C) into the HI loop of the fiber knob protein. The RGD-4C sequence has been shown to bind strongly to αvβ3 and αvβ5 integrins and enhances virus tropism in a CAR-independent fashion.^105^ Phase I trials to test these vectors in recurrent malignant gliomas patients are ongoing (NCT00805376, NCT01956734, NCT02197169, NCT03178032, and NCT03896568).^106^ Another group of oncolytic Ad vectors that can infect host cells via RGD-4C sequences and independent of CAR binding includes ICOVIR-5 and ICOVIR-7. ICOVIR-5/7 clinical trials have been initiated in cancer patients with advanced metastatic tumors (NCT01864759).^107,108^

The gene therapy vector, VCN-01, is characterized by its putative heparin sulfate glycosaminoglycan-binding site (KKTK) within the fiber shaft replaced by an RGDK motif. The vector delivers the human glycosylphosphatidylinositol-anchored enzyme, PH20 hyaluronidase, to promote viral spread in solid tumor stroma. VCN-01 has been shown to confer antitumor efficacy in patients with pancreatic cancer, especially when combined with chemotherapy (NCT02045589, EudraCT: 2012-005556-42),^109^ head and neck squamous cell carcinoma (NCT03799744), and refractory retinoblastoma (NCT03284268).

An HAd5/3 chimeric vector was created by substituting the receptor binding fiber knob domain of the HAd5 CR2-deleted vector with the knob domain of serotype 3 (Ad3), which binds to a tumor cell-enriched Ad3 receptor, circumventing CAR interaction.^110^ A phase I clinical trial of HAd5/3-Δ24 was conducted in patients with recurrent ovarian cancer.^111^ Many additional modifications have been made based on this chimeric Ad vector. For example, HAd5/3-Cox2L-D24 utilizes the tumor-specific cyclooxygenase 2 gene promoter to control E1A expression, to further enforce tumor selectivity.^112^ The vector design demonstrated safety and extended virus circulation in patients with metastatic and refractory solid tumors. The Oncos-102 drug mentioned above is also an HAd5/3 chimeric virus that targets solid tumors.^96,97^

Another tumor-selective chimeric Ad vector ColoAd1 (enadenotucirev), which was directly evolved via facilitating recombination among an array of serotypes and selecting highly potent Ad variants under stringent conditions, showed potent oncolytic activity in colon cancer cell lines (HT29). This hybrid Ad vector harbors the Ad11p (B group Ad virus) backbone and has a near complete deletion of the E3 region, a smaller deletion in the E4 region, and a chimeric Ad3/Ad11p E2B region.^113^ ColoAd1 conferred potent and unique tumor cell killing properties in multiple solid tumors.^114^ A phase l clinical trial test the synergistic effect of combining ColoAd1 with standard chemoradiotherapy has been launched to treat advanced rectal cancer (NCT03916510). Most recently, two independent phase I trials using ColoAd1 to express FAP-TAc antibody, together with an immune enhancer module (CXCL9/CXCL10/IFNα) (NCT04053283) and anti-CD40 antibody (NCT03852511), have been initiated for safety validation.

#### Challenges for Ad vector-based gene therapy

Although different types of Ad vectors are currently available for different preclinical applications, extensive study in Ad vector development have highlighted serious challenges associated with the high worldwide prevalence of pre-existing immunity against common human Ad serotypes, including HAd5. The prevalence rates for NAbs against HAd5 range from 35% in the United States to over 90% in Cote d’Ivoire.^115,116^ Circulating anti-HAd5 antibodies have been shown to significantly impair the ability of HAd5 vectors to transduce target cells and dampen the resultant adaptive immune responses.^117^ Moreover, immunogenicity and cellular toxicity continue to be major obstacles for Ad gene therapy, despite the fact that Ad vector-based vaccines and oncolytic therapies benefit from these properties. Therefore, proper control of Ad vector-mediated host innate immune responses is key for the success of these approaches.

#### The future for Ad vectors

To avoid pre-existing immunity against Ad vectors, several strategies are being employed. First, several “rare” human serotypes with low seroprevalence, such as HAd2, HAd26, and HAd35, were identified and developed into vectors to minimize pre-existing immunity. However, the ability of these vectors to induce an immune response have been shown to be less potent compared with most commonly used HAd5.^118^ In addition, various non-human Ad vectors were developed from bovine (BAd), canine (CAd), chimpanzee (ChAd), ovine (OAd), porcine (PAd), and fowl (FAd) to limit cross-reactive immunity. Chimpanzee-derived Ad vectors are most widely used, as NAbs against these in human blood circulation are significantly lower.^119^ Thus far, more than ten clinical trials using ChAd3-derived vaccine have demonstrated the safety of such replication-deficient vectors.^120^ Moreover, high-capacity HCAd vectors can achieve long-term transgene expression in vivo, since they have dampened host immune responses against viral proteins that may be residually expressed.^121^ To combat contamination by HV, self-inactivating HVs such as AdTetCre have been developed, in which a chimeric MerCreMer recombinase is regulated by a TetOn system to ensure effective elimination of HV.^122^ In addition to this advantage, the lack of viral coding sequences in HCAd genomes expands the cloning capacity to 37 kb, allowing the accommodation of site-specific nucleases such as zinc-finger nucleases (ZFNs), transcription activator-like effector nucleases, and clustered regularly interspaced short palindromic repeats (CRISPR)/Cas9 systems for genome editing.

### AAV vectors: a small vector with huge potential

#### Structure and genome

AAV was discovered in 1965 by Bob Atchison as a contaminant of Ad preparations.^123^ Due to its dependence on Ad, or any virus that can serve helper function to complete its life cycle, it is classified as a dependoparvovirus. Despite not causing any known human diseases, AAVs are remarkably well-studied in the short 40 years since their discovery. Much of our knowledge is owed to its relatively simple genome and its capacity to be experimentally manipulated in cloning plasmids.

**Fig. 4:**
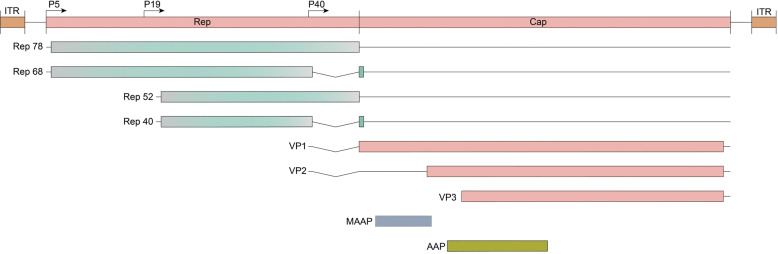
Schematic of the AAV genome and sites used for PCR screening. The AAV genome comprised four known open reading frames, *rep* (green), *cap* (salmon), *MAAP* (orange), and *AAP* (yellow). The *rep* and *cap* ORFs encode four and three isoforms, respectively. Transcription is driven by the viral P5, P19, and P40 promoters (arrows). The genome is flanked by inverted terminal repeat (ITR, cyan) sequences

As a dependoparvovirus, AAV lacks the essential genes needed for replication and expression of its own genome. These functions are provided by the Ad E1, E2a, E4, and VA RNA genes.^124,125^ The AAV genome itself, is a single-stranded DNA that houses four known open reading frames (ORFs) (Fig. 4). The first ORF encodes the four replication genes (*rep*), which are named after their molecular weights: Rep40, Rep52, Rep 68, and Rep78.^126^ The second ORF is the *cap* gene that encodes for the three viral capsid proteins, VP1, VP2, and VP3, respectively.^126^ The third and fourth are nested sub-genomic mRNAs, named the assembly-activating protein (AAP),^127^ which is involved in the shuttling of capsid monomers to the nucleolus where capsid assembly takes place; and the recently discovered membrane-associated accessory protein (MAAP),^128^ whose function is not completely understood. The 4.7-kb genome is flanked by 145-nt ITRs on both ends of the genome (Fig. 4). The ITRs serve as self-priming structures for replication, and provides the signal for Rep-mediated packaging.^129^

The AAV capsid is a *T* = 1, icosahedral, 60-mer capsid formed at a 1 : 1 : 10 ratio of VP1, VP2, and VP3 subunits, respectively. The assembled capsid is best characterized by the five-fold axis of symmetry that forms the face for rep binding and contains the five-fold pore, where DNA is inserted under rep-mediated ATPase activity; and the threefold axis of symmetry that is defined by the threefold protrusions comprising of variable loop regions V, VII, VIII, and IV.^130^ These variable loops establish the main surface epitope for antibody recognition and receptor binding. There are at least 12 natural serotypes that can be categorized into five main clades (Clades A–E) found in primates.^131^ Its sero-epidemiology is wide-spread and can be found in 50–80% of the human population, depending on the geographical region.^132^ On their own, AAVs are thought to be non-pathogenic and have yet to be concretely linked to any human diseases.

#### Infection pathway

There are more than a thousand AAV variants, falling within the five primate clades. Despite the known diversity, our knowledge of AAV’s infection pathway is based on only a few serotypes. The prevailing understanding is that serotypes bind to different, or have differential affinities to an array of primary cell surface glycoprotein receptors and secondary receptors.^133^ For example, AAV2 binds heparan sulfate proteoglycans.^134^ The identification of universal receptors has been sought after. Such efforts have discovered transmembrane proteins, such as AAVR that may act to help AAV traffic through the cell via intracellular vesicles,^135^ although not all serotypes turn out to be dependent on AAVR for transduction.^136^

Upon binding to the cell surface, clathrin-mediated endocytosis is triggered. The AAV particle is then trafficked in endosomal vesicles and are transported through late endosomal and lysosomal compartments. Due to the low pH environments of these vesicles, the capsid undergoes a conformational change to expose the VP1/2 domains.^137^ Through a mechanism that is not entirely understood, the VP1-unique region, which contains a phospholipase domain escapes the endosome or lysosome compartment, and is then shuttled into the nucleus via nuclear localization signals found on VP2. Once within the nucleus, the AAV genome undergoes second-strand synthesis to form the double-stranded genome configuration required for gene transcription. In addition, the ITRs confer intra- and inter-molecular recombination to form circular dsDNA species or concatemerized genomes.^138^ This establishes stability for the AAV genome in an epichromosomal state. Cell culture evidence and in vivo characterizations have suggested that the wild-type AAV can integrate into the genome of the host cell.^139–141^ The best known integration site in the human genome is a position in chromosome 19 called AAVS1.^140^ Although, genomic insertion is likely opportunistic with no general hotspots for wild-type AAV integration that is well-accepted.

#### AAV as a vector for gene therapy

*The vector genome*. Samulski et al.^142^ first cloned the AAV genome into expression plasmids and found that transfection of these plasmids into mammalian cell lines in the presence of Ad could produce infectious viruses. Further engineering of recombinant AAV (rAAV) demonstrated that packaging of transgenes could be achieved by gutting the genome, save for the ITR elements, and replacing it with any promoter and gene of interest. The first demonstration of an AAV vector used in humans was performed in 1995 and involved the delivery of the cystic fibrosis transmembrane regulator (CFTR) gene packaged with the AAV2 capsid (rAAV2-CFTR), into a patient with cystic fibrosis.^143^ Since this first demonstration, multiple vector designs have been reported. The design of vectors in a way is an art form, the reasons for which is manifold. The main consideration for AAV vector design is that the wild-type genome is ~4.7 kb in size. Thus, vectors based on them are irrevocably limited to a ~5 kb capacity. All components needed for proper expression therefore need to be abbreviated/truncated/minimized to fit into the small capsid. Alternatively, strategies that exploit ITR-mediated recombination have produced dual-vector systems that can express “oversized” transgenes, by way of transcript splicing across intermolecularly recombined ITRs from two complementary vector genomes.^144–146^ Other means of promoting vector-size expansion through vector recombination by homology,^147^ RNA trans-splicing,^148^ or protein “*trans*-splicing” via split intein designs^149,150^ have also been developed.

The promoters used in AAV vectors, also known as regulatory cassettes, to drive transgene expression are also an important aspect of vector design. As contemporary AAV serotypes or engineered capsids have the capacity to transduce multiple tissue/cell types, cell-type-specific transgene expression is by and large controlled at the level of gene transcription. Designing tissue-specific promoters is the main approach for improving on-target tissue expression. In addition, not only do regulatory cassettes need to be tissue-specific, they must be powerful enough to confer therapeutic levels of transgene expression. For example, the use of muscle-specific regulatory cassettes based on the muscle creatine kinase gene has been employed for muscle gene therapy treatments,^151^ such as Duchenne muscular dystrophy (DMD) and limb-girdle muscular dystrophy (LGMD). To meet the challenges of the small packaging size, bidirectional vectors have also been employed for delivery of dual therapeutic gene cassettes. An example of this is the bidirectional chicken β-actin ubiquitous promoter that drives the simultaneous expression of the hexosaminidase α- and β-subunits of the HexA enzyme, the two respective genes involved in Tay-Sachs and Sandhoff diseases.^152^

Other non-promoter vector design strategies to control transgene expression exploits the host cell’s response to foreign pathogens. A standing challenge for AAV-mediated gene therapy is how innate immunity plays a role in muting transgene expression. There is evidence that Toll-like receptor 9 (TLR9) activation and MyD88 can be activated by the presence of unmethylated CpG dinucleotides.^153^ One straightforward way to circumvent this challenge is to deplete the vector genome of CpGs by codon optimization and mutation of promoter elements;^153^ although, overall expression can be affected.^154,155^ Other methods for reducing the activation of the immune system is to employ the cell’s own RNA interference machinery to inhibit expression in antigen-presenting cells (APCs) by inserting binding sites for microRNAs (miRNAs) that are highly expressed in these cell types,^156^ such as miR142, into the vector genome. This approach effectively eliminates MHC class I-mediated antigen presentation in APCs, hence suppressing CD8+ T-cell activation.^157–159^ Alternatively, designs to include DNA sequences that are derived from human telomeres have been reported to inhibit TLR9 activation and reduce rAAV-associated immune responses.^160^

One frontier that has yet to be fully explored is the rational engineering of ITR regions.^161^ Currently, the only widely used modification to this essential element is to make one ITR unresolvable during replication. By mutating one of the ITRs so that Rep can no longer nick the terminal resolution sequence, a double-stranded genome is packaged into capsids, yielding vectors termed “self-complementary” AAVs (scAAVs).^162,163^ This modification allows for the bypass of second-strand synthesis, the rate-limiting step for rAAV transduction. The scAAV platform significantly enhances transduction onset and efficiency. The drawback is that the maximum genome size is effectively halved, restricting the packaging limit of the vector. In addition, scAAV genomes can trigger a stronger innate immune response to the transgene compared to single-stranded vectors.^164–166^ Aside from manipulating how the AAV genome is packaged, is has long been evidenced that the ITR harbors promoter function.^167^ The extent by which ITRs can impact transgene expression is not well-studied. However, new evidence suggests that the ITRs of different serotypes can confer differential promoter activity.^168^ Furthermore, there is evidence that the ITRs can produce transcripts on the minus strand, independent of a classical promoter on the reverse strand.^169,170^ The result of such action is the formation of double-stranded RNAs, which can trigger the host innate immune system. Our lack of full understanding for the AAV vector transduction pathway, and ways in which the ITRs can serve to impact vector genetic fate, has limited our ability to modulate the very last viral elements still residing in current vectors used for gene therapy.

*Capsids*. The advancement of AAV to expand the capsid toolbox has been the focal point for improving AAV vectors in recent years. The capsid is not only critical for the recognition of cell surface proteins that impact tissue/cell tropism,^133^ but also serves as the epitope for antibody recognition,^171^ functions as the substrate for phosphorylation (either prior to or during viral egress from the late endosome for proteasomal degradation),^172^ and has been shown to remain bound to the genome to impact second-strand synthesis and transcription in a cell-type-specific manner.^173,174^ As AAVs cannot be easily engineered with capsid proteins from other non-related viruses to yield chimeric vectors with modified properties (pseudotyped), work in the past two decades has focused on developing new capsids with improved properties via alternative approaches. There are currently four main methods for capsid discovery: (1) vectorization of AAV capsids from natural isolates; (2) rational design of capsids using pre-existing capsid as scaffolds; (3) directed evolution of capsid libraries generated by error-prone PCR and/or shuffling of pre-existing capsids with desired properties; and (4) in silico approaches involving the use of computational tools to design novel capsids not observed in nature. We will highlight some of the milestones met in these four categories.

i. Natural isolates. Serotypes AAV1 and AAV2 were first discovered from tissue culture. Afterwards, AAV serotypes were isolated from a range of mammalian species and tissue types. Between 1965 to 2001, only a handful of AAVs, mostly from human clinical samples, were derived from viral isolates, vectorized, and tested. Following these efforts, which demonstrated that each serotype had unique tropism profiles, larger studies to isolate AAVs from human and non-human primate tissues were accomplished by using PCR- amplification of proviral *cap* sequences. At the end of 2009, more than a hundred variants across six different clades were discovered.^131,175,176^ Although only a few research groups continue to screen natural variants as potential gene therapy vectors, they are still the predominant capsids used in clinical studies today.

ii. Rational design. The engineering of pre-existing capsids by rational design is an efficient means of developing new capsids with specific properties.^177^ Rational design approaches have produced some capsids with beneficial characteristics that are improved over natural variants. Such strategies began with the grafting of peptide sequences known to bind surfaces of target tissues onto the surface of the capsid.^178^ The latest achievement using such a strategy was the development of bone-homing capsid named AAV9.DSS-Nter.^179^ However, the generation of functional capsids through these means are in general difficult. Part of the reason behind this difficulty is that the AAV capsid and it’s role during the viral life cycle is not completely understood, i.e., changes on the capsid may theoretically improve binding of AAV to specific cell receptors, but may negatively impact other aspects, such as vector production or cellular trafficking.

iii. Directed evolution. Iterative selection of randomly mutated capsids in cell culture or in animals, coupled with next-gen sequencing strategies have given researchers a manageable means of developing new capsids with defined properties.^177^ Randomized mutations can be achieved by error-prone PCR^180^ or by the generation of chimeric capsids (capsid shuffling) from the primary sequences of pre-existing serotypes.^181–183^ The most noteworthy examples of evolved capsids are derived from platforms based on the insertion of short, randomized peptide sequences onto the threefold protrusions, which are highly amenable for capsid manipulation.^184^ Among the first in this class of capsids are AAV2.7m8 and AAVPHP.B.^185,186^ The AAV2.7m8 capsid was derived by the insertion of a random seven-amino acid sequence into loop VIII of the AAV2 capsid and iterative rounds of random mutagenesis and selection of positively transduced photoreceptor cells. The development of high-throughput sequencing approaches in the early 2000s marked not only the capacity to profile the transcriptome and epigenetic marks on a whole-genome scale but also allowed for AAV investigators to expand screening depth to test millions of variants at once. Unfortunately, these screening methodologies are limited in that they can only evolve capsids that perform well in the systems they are screened in. For example, the discovery of AAV.PHP.B, which was screened for its ability to traverse the blood–brain barrier (BBB) in C57BL6 mice, produced a capsid that exhibited strain-specific differences.^187^ Nonetheless, this work was able to reveal the species- and strain-dependent *LYS6A* gene as the critical receptor that permits AAV.PHP.B to traverse the BBB.^188^ Additional capsids have thus far been evolved that do not rely on *Lys6A* host expression, such as AAV-F.^189^ Further improvements to these methodologies are ongoing and they offer a rapid means of developing new capsids for specific targets.

iv. In silico approaches. The use of computational approaches to develop novel AAV capsids for gene therapy is a relatively new concept.^177^ Within the past few years, this approach is best exemplified by the discovery of Anc80, a capsid whose sequence is predicted to be the “ancestral” capsid by phylogenetic connectivity between contemporary capsids. Importantly, Anc80 has shown strong tropism to the mouse liver, muscle, retina, and cochlea.^190,191^ The evaluation of germline endogenous viral elements (EVEs) also represent attempts to reconstruct novel ancestral capsids with the potential for vectorization.^192^ By using bioinformatics approaches to identify and analyze EVEs among several marsupial species, “fossilized” viral integration events within host DNA have been uncovered. Recently, the single-amino acid substitution across the entire AAV2 *cap* ORF in combination with machine learning approaches have been used to further increase the depth of variants that can be queried for improved vector performance. The use of machine learning approaches truly represents a new frontier in the gene therapy field. Computer-assisted structure–function analyses has great potential for producing capsids sequences not seen in nature. These tools are developed to consume and integrate large amounts of data on the performance of capsids with single or multiple amino acid changes (production and transduction efficacies), to ultimately derive novel capsids with desired properties. These means can potentially bypass the iterative steps required for directed evolution and can overcome the trial and error process attributed to rational design approaches. Machine-guided strategies have already demonstrated their utility by uncovering a previously unknown ORF encoding for the *MAAP* gene.^128^

#### The nuances of AAV vector biology

AAV vectors have been universally recognized as versatile vectors for gene therapy. Much of this is owed to their wide-ranging tropism profiles, even by the contemporary serotypes. As a result, most AAV vector platforms have been developed to target diseases of the central nervous system, the eyes, liver, heart, and muscle. Similar to their wild-type counterparts, AAV vector genomes undergo circularization via ITR recombination to form stable and persistent episomal configurations that can be detected well beyond 10 years following administration in non-dividing, terminally differentiated cell populations.^193–195^

Compared to other current viral vectors, rAAVs are accepted as the least immunogenic, with much less vector-related toxicity. In contrast to Ad vectors, AAVs are ideal for their lack of transgene immunity when expressing self-antigens and confer relatively low innate immunity and viral immunity within a broad dose range.^196,197^

One final nuance for AAV vector research is that assessing AAV vector performance is not ideal with in vitro models. Typically, transduction of rAAVs in vivo does not tend to reflect what is observed in vitro. Furthermore, the field is increasingly aware that results gleaned from small animal models may not necessarily be reproduced when tested in human trials. This failure has led to the necessity for researchers to carry out studies in non-human primate models such as cynomolgus or rhesus macaques before clinical trials are realized.

#### Production platforms

Although AAV-based vectors have shown great promise as gene therapy drugs, one of the limitations for rAAVs is achieving scalable and economical production of vectors that are free of impurities. Since the initial vectorization studies to produce single-strand rAAVs in the 1980s, clinically relevant advancements in AAV vectorology have been intermittent. The scAAV platform is sometimes referred to as the second generation of rAAVs, but they were developed nearly 20 years ago. In contrast, vector production platforms have undergone multiple advancements. Since both single-stranded AAV and scAAV vectors are absent of all viral components save for the ITRs, the universal strategy for different production schemes is to achieve optimal *trans* expression of obligatory factors required for packaging AAV. Knowing that impurities continue to be a burden for generating high-quality vectors, efforts to streamline and improve vector yields have been the focus for improving manufacturing practices in recent years. Currently, vector production schemes for clinical use have yet to adopt strategies that are completely cell free.

*Triple transfection: HEK293*. Preclinical/research-grade AAVs are still predominantly produced by the standard triple-transfection method in HEK293 cells. Typically, this employs the co-transfection of the cis plasmid containing the transgene cassette of interest, flanked by ITRs; the trans plasmid, which houses the *rep* and *cap* genes, and the Ad helper plasmid, which provides the E2a/b, E4, and VARNA genes. As HEK293 cells are transformed by Ad,^198^ they express the essential *E1a* and *E1b* genes. These cells are typically incubated as semi-adherent cultures and reach maximum productivity at 2–3 days post transfection, losing viability overtime.^199^

*Mammalian stable cell lines*. Stable cell lines that constitutively express vector components have been developed to streamline the production process. These platforms free the need to deliver, for example, capsid expression cassettes and/or helper genes on a plasmid. They only require the delivery of the vector genome to be packaged. Clark et al.^200^ first developed a HeLa-based producer cell line in the mid-1990s, by cloning *rep*/*cap* and the rAAV vector genome into an expression plasmid, and then using it to stably transfect HeLa cells. Such a cell line would only require infection by wtAd5 to trigger high-yield vector production. One obvious limitation of this strategy, is that a cell line must be produced for every vector design. Alternatively, hybrid rAAV/rAd vectors were developed to deliver both the transgene vector and adenoviral elements to provide robust *rep-cap* expression from stably integrated AAV genes in HeLa cell lines. These platforms yield high-titer vector for large-scale production needs.^201,202^ Producer cell lines derived from A549, a lung-epithelial carcinoma cell line, have also been developed.^203^ Other vector-based production methods such as HSV vectors have been engineered to deliver within them AAV vector components into baby hamster kidney fibroblast cells (BHK21).^204^ These platforms are great for production scale-up and enables the generation of clinical-grade vectors. In addition, the use of stable cell lines limits the auxiliary components necessary for production and removes sources of contamination, like adventitious viruses or plasmid contaminants originating from DNA preparation steps. Unfortunately, the concern with using immortalized or transformed cell lines is that the elements that confer their characteristics can be packaged into the vectors as impurities, raising the possibility of genotoxicity in the host, as well as potentially harmful carryover helper viral impurities. Furthermore, unlike the HEK293-based platforms, these stable cell lines lack E1a/E1b expression and therefore require replication-competent Ad helper and/or Ad-AAV (replication-defective) hybrid virus infection. Thus, many methods have been developed to determine whether potential oncogenes or transforming factors end up in manufactured AAVs. Most recently, SSV-seq, AAV-GPseq, and Fast-seq approaches have all been developed to address the relative abundance of mispackaged nuclear material that winds up in preparations.^205–207^

*Baculovirus/Sf9*. An alternative production scheme to mammalian cell lines is the use of baculovirus expression vector (BEV) system in *Spodoptera frugiperda* insect cells (Sf9). This involves the generation of recombinant baculoviruses: one BEV carrying the AAV vector genome, flanked by ITRs, and in another, the *rep* and *cap* genes. The BEVs are in turn used to infect Sf9 cells. Since the baculovirus provides helper function,^208^ a single BEV system in conjunction with Sf9 cell lines that stably express *rep*, can be employed for flexible and high-titer large-scale vector production.^209^ Interestingly, a report that vectors produced from Sf9 cells are differentially posttranslationally modified, as compared to those generated from human- or mammalian-derived cell lines raised question as to whether differences in production schemes may impact tropism and transduction efficacies,^210^ although further work in this area is necessary to cross-validate these findings. Nonetheless, BEV/Sf9 systems exhibit reduced encapsidation of contaminating DNAs^205^ and therefore remain an attractive production strategy for large-scale, clinical-grade vectors.

#### Therapeutic AAV vectors and commercial availability

To date, there are have been three AAV-based gene therapy drugs worldwide that have made it to the commercial market. Although they symbolize the things to come for these promising platforms, two major challenges remain: cost of treatment and safety. Manufacturing represents a third challenge. However, the demand for these drugs has yet to reach levels in which manufacturing has created significant bottlenecks. Although manufacturing limitations can still slow clinical trials, most of these drugs are currently developed towards monogenic diseases, which belong to the category of rare diseases and the patient population eligible for trials remains relatively low.

Glybera (alipogene tiparvovec) was the world’s first AAV-based gene therapy to gain regulatory approval for commercialization from the European Medicines Agency in 2012.^211^ Glybera is an AAV1-based gene therapy that delivered lipoprotein lipase (LPL) to patients with LPL deficiency.^212^ It was also infamous for gaining the moniker of “the world’s first million-dollar drug.” Due to low demand and its high cost, Glybera was withdrawn from European markets and has yet to gain approval in other countries. Altogether, only four patients outside of clinical trials ever received the drug, three of which payed only €1 to expend the remaining unused stocks.

Luxturna (voretigene neparvovec-rzyl) was the second AAV drug that gained commercial approval in 2017—first by the U.S. Food and Drug Administration (FDA). Luxturna is an AAV2-based vector that delivers the retinoid isomerohydrolase RPE65,^213^ the effected gene in Leber’s congenital amaurosis, which causes progressive blindness. On its launch, the drug held a price tag of $425,000 per eye.

Zolgensma (onasemnogene abeparvovec) is the third and the most recently approved AAV drug, developed for the treatment of spinal muscular atrophy (SMA).^214^ It gained approval in the United States and Japan in 2019, and received approval in the EU in 2020. Zolgensma is a one-time gene therapy that delivers the survival motor neuron (SMN1) transgene to replace the non-functional *SMN1* gene mutated in patients with SMA. During its launch, it was marketed at $2.125 million per dose—currently, the most expensive medication in the world.

#### Clinical trials

With the approval of the first AAV-based drugs and continued promising clinical data across the field, several gene therapy platforms have sprung forth and are currently under investigation in the clinic. As a result, there have been over 200 clinical trials based on AAV worldwide. For many trials, enrollment is limited to only a few individuals and the selection criteria are many times very restricted. Combined with the fact that many current gene therapy strategies are presently only developed for rare diseases, the advancement of these new drugs are slow. The FDA and National Institutes of Health are now recognizing that the number of eligible patients is limited and many AAV treatments are for lethal diseases with no alternative options. Therefore, many combined phase I/II or II/III trials have been granted. Table 2 summarizes all current AAV-based clinical trials. The following clinical trials highlight some of the most noteworthy outcomes.

**Table 2:** A selection of ongoing clinical trials employing AAV vectors

Condition	Intervention	Sponsor	Trial stage	Identifier
AADC deficiency	AADC	Krystof Bankiewicz UCSF	Phase I	NCT02852213
	AADC	National Taiwan University Hospital	Phase II	NCT02926066
Batten disease (CLN2)	CLN2	Weill Cornell	Phase I/II	NCT01414985
Batten disease (CLN6)	CLN6	Nationwide Children’s Hospital	Phase I/II	NCT02725580
MPS-IIIB	NAGLU	UniQure	Phase I/II	NCT03300453
Parkinson disease	AADC	Jichi Medical University	Phase I/II	NCT02418598
	GDNF	NINDS	Phase I	NCT01621581
	Neurturin	Sangamo	Phase I/II	NCT00985517
	AADC	Voyager	Phase I	NCT03065192
SMA	SMN	AveXis	Phase III	NCT03461289
GAN	GAN	NINDS	Phase I	NCT02362438
Achromatopsia	CNGB3	AGTC	Phase I/II	NCT02599922
	CNGB3	MeiraGTx	Phase I/II	NCT03001310
Choroideremia	REP1	Nightstar	Phase III	NCT03496012
	REP1	Spark	Phase I/II	NCT02341807
	REP1	STZ Eyetrial	Phase II	NCT02671539
	REP1	University of Oxford	Phase II	NCT02407678
LCA	RPE65	Spark	Phase III	NCT00999609
	RPE65	MeiraGTx	Phase I/II	NCT02781480
LHON	ND4	GenSight	Phase III	NCT03293524
	ND4	John Guy University of Miami	Phase I	NCT02161380
RP (RLBP1)	RLBP1	Novartis	Phase I/II	NCT03374657
Wet AMD	Anti-VEGF antibody	Regenxbio	Phase I	NCT03066258
	Anti-VEGF protein	Adverum Biotechnologies	Phase I	NCT03748784
X-Linked RP	RPGR	AGTC	Phase I/II	NCT03316560
	RPGR	MeiraGTx	Phase I/II	NCT03252847
	RPGR	Nightstar	Phase I/II	NCT03116113
X-linked retinoschisis	RS1	AGTC	Phase I/II	NCT02416622
	RS1	NEI	Phase I/II	NCT02317887
Crigler–Najjar syndrome	UGT1A1	Audentes	Phase I/II	NCT03223194
	UGT1A1	Genethon	Phase I/II	NCT03466463
FH (homozygous)	LDLR	University of Pennsylvania	Phase I/II	NCT02651675
GSD1a	G6PC	Ultragenyx	Phase I/II	NCT03517085
Hemophilia A	FVIII	Shire	Phase I/II	NCT03370172
	FVIII	Bayer	Phase I/II	NCT03588299
	FVIII	BioMarin	Phase III	NCT03392974
	FVIII	Sangamo	Phase I/II	NCT03061201
	FVIII	Spark	Phase I/II	NCT03003533
	FVIII	UCL	Phase I	NCT03001830
Hemophilia B	FIX	Shire	Phase I/II	NCT01687608
	FIX	Pfizer	Phase II	NCT02484092
	FIX	Pfizer	Phase III	NCT03587116
	FIX	Sangamo	Phase I	NCT02695160
	FIX	St. Jude Children’s Research Hospital	Phase I	NCT00979238
	FIX	UniQure	Phase III	NCT03569891
	FIX	UCL	Phase I	NCT03369444
	FIX	Freeline Therapeutics	Phase II/III	NCT03641703
MPS-I	ZFN1, ZFN2, IDUA donor	Sangamo	Phase I	NCT02702115
MPS-II	ZFN1, ZFN2, IDS donor	Sangamo	Phase I	NCT03041324
MPS-IIIA	SGSH	LYSOGENE	Phase II/III	NCT03612869
MPS-VI	ARSB	Fondazione Telethon	Phase I/II	NCT03173521
OTC deficiency	OTC	Ultragenyx	Phase I/II	NCT02991144
A1AT deficiency	A1AT	UMMS	Phase I	NCT00377416
CMT1A	NTF3	Nationwide Children’s Hospital	Phase I/II	NCT03520751
DMD	Microdystrophin	Nationwide Children’s Hospital	Phase I/II	NCT03375164
	Mini-dystrophin	Pfizer	Phase I	NCT03362502
	Microdystrophin	Solid Biosciences	Phase I/II	NCT03368742
	Microdystrophin	Sarepta Therapeutics	Phase II	NCT03769116
LGMD, type 2E	LGMD2E	Sarepta Therapeutics	Phase I/II	NCT03652259
Dysferlinopathy	DYSF	Nationwide Children’s Hospital	Phase I	NCT02710500
HIV infections	PG9 antibody	International AIDS Vaccine Initiative	Phase I	NCT01937455
	VRC07 antibody	NIAID	Phase I	NCT03374202
Pompe disease	GAA	Actus Therapeutics	Phase I/II	NCT03533673
	GAA	University of Florida	Phase I	NCT02240407
X-linked MTM	MTM1	Audentes	Phase I/II	NCT03199469

*A1AT* α1 antitrypsin, *AADC* aromatic l-amino acid decarboxylase, *AGTC* Applied Genetic Technologies Corporation, *AMD* age-related macular degeneration, *ARSB* arylsulfatase B, *CLN2* neuronal ceroid lipofuscinosis type 2, *CMT1A* Charcot–Marie–Tooth disease type 1A, *CNGB3* cyclic nucleotide-gated channel-β3, *DMD* Duchenne muscular dystrophy, *DYSF* dysferlin, *FH* familial hypercholesterolemia, *FVIII* factor VIII, *G6PC* glucose-6-phosphatase catalytic subunit, *GAA* α-glucosidase, *GAN* gigaxonin, *GDNF* glial cell line-derived neurotrophic factor, *GSD1a* glycogen storage disease type 1a, *LCA* Leber congenital amaurosis, *LDLR* low-density lipoprotein receptor, *LHON* Leber hereditary optic neuropathy, *mAb* monoclonal antibody, *MPS* mucopolysaccharidosis, *MTM* myotubular myopathy, *NAGLU* *N*-α-acetylglucosaminidase, *ND* not disclosed, *ND4* NADH-ubiquinone oxidoreductase chain 4, *NEI* National Eye Institute, *NIAID* National Institute of Allergy and Infectious Diseases, *NINDS* National Institute of Neurological Disorders and Stroke, *NTF3* neurotrophin 3, *OTC* ornithine transcarbamylase, *REP1* RAB escort protein 1, *RLBP1* retinaldehyde-binding protein 1, *RP* retinitis pigmentosa, *RPE65* retinal pigment epithelium-specific 65 kDa protein, *RPGR* retinitis pigmentosa GTPase regulator, *RS1* retinoschisin 1, *SGSH* *N*-sulfoglucosamine sulfohydrolase, *SMA* spinal muscular atrophy, *SMN* survival of motor neuron, *UCL* University College London, *UCSF* University of California San Francisco, *UGT1A1* UDP glucuronosyltransferase family 1 member A1, *UMMS* University of Massachusetts Medical School, *VEGF* vascular endothelial growth factor, *ZFN* zinc-finger-containing protein.

*Age-related and diabetic macular degeneration (AMD)*. ADVM-022 is an intravitreal injection of an AAV vector carrying aflibercept, an anti-VEGF fusion protein that blocks blood vessel growth and leakage related to AMD and diabetic retinopathy. What makes this gene therapy stand out is the use of AAV2.7m8, the evolved capsid that is strongly tropic to photoreceptors.^185^ Current phase I clinical trials aim to assess safety and tolerability of a single intravitreal injection in patients with AMD, correction of visual acuity, and the elimination of anti-VEGF rescue injections (NCT03748784). The most recent and positive outcomes from patients receiving treatments showed mean best corrected visual acuity improvements (+6.8 letters) and central subfield macular thickness reduction (−137.8 μM), indicators of disease reversal.^215^

*Muscular dystrophies*. The fight against DMD has a long history and, thanks to awareness efforts throughout the decades, groundbreaking research has resulted in successful treatments of the disease via AAV-based gene therapy.^216^ DMD is a fatal X-linked neuromuscular genetic disease that occurs in ~1 in every 3500–5000 males worldwide. DMD is caused by mutation(s) in dystrophin, a structural gene that provides tensile strength and integrity to striated muscles, and is one of the largest proteins in the human body. As a result of many years of work, several groups have developed platforms with clinical trials currently underway.

SRP-9001 is an AAVrh.74 capsid-based vector that harbors a shortened form of dystrophin. The discovery of microdystrophin, whose shortened design was inspired by the mutant form of the dystrophin gene observed in Becker muscular dystrophy, is the basis of the vector’s success.^217^ As AAV is limited in its packaging capacity, the relatively short transgene allows for packaging of the transgene along with the striated muscle-specific regulatory cassette called αMHCK7, a combined α-myosin heavy chain enhancer and version 7 of the creatine kinase promoter.^151^ The treatment confers systemic and robust therapeutic transgene delivery to skeletal and cardiac muscles. A similar gene therapy strategy to tackle LGMD (SRP-9003) has also implemented. SRP-9003 delivers β-sarcoglycan—a gene when missing, causes LGMD Type 2E. Trials to test these gene therapy platforms are under the respective clinical trial identifiers NCT03769116 and NCT03652259.

Despite the promise of some DMD gene therapy trials, others have been met with challenge. SGT-001 is also an AAV vector packaging microdystrophin. Although clinical outcomes for trials testing SGT-001 (NCT03368742) showed that 50–70% of muscle fibers express the therapeutic transgene and conferred a decline in muscle damage, some patients had adverse effects, where at least one patient had an immunological response to the gene therapy.^218^ Albeit the adversity was resolved, the FDA’s decision to halt trials are a reflection of its dedication to ensuring that trials are safe for the patients. Similarly, patients who were systemically administered with high doses of PF-06939926 (NCT02310763) experienced transient and/or acute renal impairment, accompanied by activation of the complement system.^219^

*Hemophilia*. The gene therapy field for hemophilia, a family of severe blood clotting diseases, has long had its ups and downs. The numerous human trials in the mid-1990s to the early 2000s reflected the diversity of strategies aimed at correcting this blood clotting disease. In fact, AAVs, retrovirus, and Ad vectors were all explored. The first AAV-based hemophilia strategies aimed to target muscle tissues to act as biofactories for expressing factor IX (F.IX) treat hemophilia B^220^ showed no toxicity and sustained transgene presence beyond multiple years post administration. However, these studies also demonstrated that the therapeutic dose via intramuscular injections could not be achieved by muscle-targeted transgene expression without larger vector doses or clinically impractical injection regimens.^221,222^ In a milestone trial, delivery of AAV-F.IX to the liver showed successful transduction to human hepatocytes. However, this result was also met by immunological responses to capsid protein and subsequent decline in circulating F.IX levels, showing that immunotoxicity was a critical barrier for therapeutic vectors.^223^ Nearing the end of 2017, AAV delivery of FVIII to the liver with AAV5^224^ and a high-activity F.IX variant (F.IX-Padua), which exhibits eight times more activity than the normal protein, also targeting the liver,^225^ were demonstrated in trials. There are currently at least 12 ongoing clinical trials that are being pursued by 5 pharmaceutical companies. Many of these are in phase II/III stages and thus show promise for commercialization, namely fidanacogene elaparvovec, developed to treat hemophilia B; SPK-8011, a gene therapy for hemophilia A; Valrox (valoctocogene roxaparvovec or BMN 270), a gene therapy to treat hemophilia A; and AMT-061, a gene therapy for hemophilia B, are currently being tested in phase III clinical trials (NCT03392974 and NCT03569891). FLT180a is also in a phase II/III study with hemophilia B patients in the U.K (NCT03641703).

*X-Linked myotubular myopathy (XLMTM)*. Recently, a clinical trial involving high-dose administration of AT132 (NCT03199469), an investigational gene therapy product candidate for XLMTM, led to the deaths of three patients who had progressive liver dysfunction, characterized by hyperbilirubinemia.^226–228^ The patients were notably of older age, heavier weight, and had prior histories of hepatobiliary disease. Nevertheless, this unfortunate outcome signals how important it is to determine safe, therapeutic doses. It is a continuing challenge for researchers and clinicians to advance their therapeutic strategies to human patients, knowing that there are risks involved. Still, these trials are very much needed.

#### Challenges

The growing number of clinical trials using rAAVs is a positive sign that these vector-based gene therapies hold a lot of promise; yet, there are still quite a lot of challenges that the field has yet to address. Immunogenicity towards the vector remains the largest challenge for AAV-based gene therapies. In fact, the immune system will always be a major barrier for any gene therapy approach. Thus far, adaptive immunity to the capsid and the foreign transgene represents major factors for decreased efficacies. Although the single administration aspect and sustainability of AAV gene therapy mean that the need for redosing can be avoided, patients that have been exposed to AAV serotypes that a gene therapy is based on, will have a high-chance of presenting NAbs against the vector capsid.^171^ The goal of discovering new capsids seeks to partially address the presence of NAbs against serotypes commonly found in populations. For circumstances where tailoring the vector platform to the patient is not possible, plasmapheresis is a plausible means of removing anti-AAV antibodies from the bloodstream^229^ or pre-treatment with IgG-cleaving endopeptidases such as imlifidase (IdeS) can nonspecifically reduce IgG antibodies from the sera of patients.^230^ IdeZ, a homolog of IdeS, isolated from *Streptococcus zooepidemicus*, demonstrated efficacious removal of capsid NAbs in non-human primate (NHP) studies.^231^ Adaptive immunity towards the transgene may be more straightforward to address. As mentioned above, detargeting from professional APCs, such as dendritic cells with tissue-specific promoters and the design of miRNA binding sites for miR142 to silence transgene expression, can circumvent adaptive immunity.^157–159^

Mechanisms for innate immunity have been well-described in response to viruses, but exploration of innate immune response towards AAV vectors is understudied. Serotype-specific responses have been observed and can be directly addressed. Namely, differences in receptor binding between AAV2 and AAV8, whereby AAV2’s preference for heparin proteoglycans may confer higher T cell response, may lead to strategies to mitigate immunity via rational design.^232^ In addition, evidence is accumulating for the possibility that the AAV vector genome can elicit an innate immune response,^233^ necessitating an area of research that is critically needed. TLR9 detection of methylation-free CpG dinucleotides is one means by which cells can identify foreign DNA. Other foreign DNA- and RNA-sensing mechanisms, involving RIG-I (*Ddx58*), CGAS (*Mb21d1*), and STING (*Tmem173*), may also play roles.^234^

Finally, a challenge that must be confronted is managing the right treatment doses, which may be at the heart of the strong immunological responses and subsequent toxicities seen in recent trials. Although AAV-based gene therapy for SMA has been proven to be safe, interpatient differences may impact the response. In a preclinical study in piglets and non-human primates, where supraphysiological transgene expression was achieved, proprioceptive deficits and ataxia was observed, and was attributable to high transduction of the dorsal root ganglia (DRG) and subsequent toxicity.^235^ In fact, a recent meta-analysis of 33 non-clinical studies encompassing 256 NHPs showed that the majority of animals administered via cerebrospinal fluid (CSF) exhibited DRG pathologies.^236^ Using miRNA-detargeting can reduce toxicity.^237^ These studies and others indicate that further evaluation of the appropriate routes of administration, capsid choice, and vector genome designs are still needed, even for approved drugs.

#### The future for AAV vectors

Despite some setbacks, AAV vectors still hold huge promise for correcting disease. Now with the preclinical successes seen with CRISPR/Cas systems, which allow programmed editing nearly anywhere within the genome, the potential for AAVs seems limitless.^238^ However, every approach has their downsides. AAV vectors have always been considered relatively safe, yet recent demonstration that AAV vector genomes carrying CRISPR components can integrate into the host cell genome at the site of double-strand breaks^239^ has raised concerns over their efficacies and long-term safety profiles. In fact, in a 10-year follow-up study in six dogs receiving gene therapy vector for F.VIII, vector genomes were found to be stably integrated into the host genome and has re-raised concerns for oncogenic integration of AAV.^240^ Despite the overall positive safety track record for AAV treatments in humans, continued efforts in gauging the long-term and short-term safety for AAV vectors are certainly warranted.

### Lentivirus vectors: a robust vector for genetically modified cell therapies

#### Structure and genome

Lentiviruses constitute a genus of the retroviridae family. Retroviruses are spherical, enveloped, single-stranded RNA viruses that are ~100 nm in diameter.^241,242^ The lentiviral particle encapsidates two sense-strand RNAs that are bound by nucleocapsid proteins. The particle also contains reverse transcriptase, integrase, and protease proteins. Retroviruses can be classified into simple or complex viruses, based on their genome organization. Gammaretroviruses are an example of simple retroviruses, whereas the HIV-1, a lentivirus, is an example of a complex retrovirus. The 9.7 kb HIV-1 genome is flanked by 5′- and 3′ LTRs, which are integral to viral genome replication. The LTR is composed of U5 and U3 sequences that are unique to the 5′ and the 3′ termini of the viral genome, respectively, and R, which is a common sequence at both ends. The *cis*-acting sequence, also known as *psi*, resides just outside of the LTR and is pivotal for signaling viral genome encapsidation.^243,244^ Retroviruses have common essential core protein genes, such as *gag*, *pol*, and *env*. Although the *gag* gene encodes for the protective capsid and matrix proteins, the *env* gene carries information for transmembrane and envelope glycoproteins that dictate the virus’ cellular tropism.^245^ Reverse transcriptase and integrase are transcribed from the *pol* gene. As part of an intricate regulatory machinery that facilitates viral replication, lentiviruses have additional genes called *tat* and *rev*. Tat supports transcriptional activation and RNA polymerase II elongation by binding adjacently to the LTR.^246^ Rev orchestrates nuclear export of spliced and un-spliced viral RNA by binding to a motif in the *env* gene region.^247^ Lentiviruses, such as HIV-1, have an additional set of auxiliary genes (*vif*, *vpr*, *vpu*, and *nef*), which elevate the viral titer and pathogenesis of the virus.^248,249^

#### Infection pathway

The entry of infectious lentiviral particles to the host cell is mediated by interactions between glycoproteins anchored on the outer envelope and a specific cell receptor. Successful binding to cell surface receptors prompts a series of events that lead to the fusion of the viral particle lipid bilayer and the cell, and subsequent unloading of the viral genetic cargo into the cytoplasm.^250–252^ Lentiviral DNA integrates into the host genome in a non-random manner with preference for transcriptionally active sites.^253–255^

#### Lentiviruses as vectors in gene therapy

Lentiviral vectors have several features that make them amenable to transgene delivery for therapeutic purposes. Lentiviral vectors are integrating vectors that permit long-term transgene expression. They have a packaging capacity of up to 9 kb.^256^ High-level expression of multiple genes may be a requisite for achieving a therapeutic outcomes for certain diseases. Employing two separate vectors carrying co-dependent transgenes may not be an optimal solution, as successful transduction of multiple viral vectors to the same cell is not efficient. Lentiviral vectors are demonstrated to have the ability to express multiple genes from a single vector.^257–259^ Lentiviral vectors can transduce postmitotic and quiescent cells, whereas other retrovirus-based platforms, such as gammaretroviral vectors, require active cell division for successful infection. Although quiescent cells are mostly recalcitrant to infection, partly as a result of the innate immune response,^260^ stimulation of mitotic entry can facilitate viral transduction.^261–264^ Depending on the viral vector design, lentiviral vectors elicit relatively weak immune responses.^265,266^

**Fig. 5:**
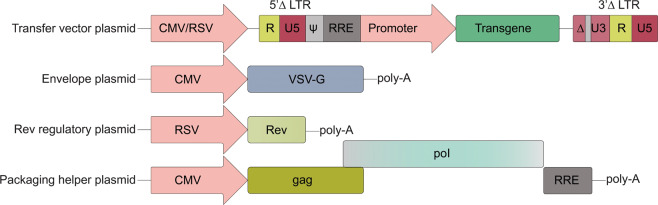
Third-generation HIV-1-based lentiviral vector design. The third generation of lentiviral vectors are produced using four plasmids. The first plasmid has a construct carrying the gene of interest driven by a desired promoter flanked by long terminal repeats (LTRs). Both 5′ and 3′ LTRs in wild-type HIV-1 is composed of U3, R, and U5 sequences. The U3 sequence constitutes promoter/enhancer elements. Part of the U3 sequence in the 3′-LTR is deleted, and the entire U3 sequence within the 5′-LTR is replaced by a strong viral promoter, such as CMV. The psi (ψ) packaging signal is followed by the *rev* response element (RRE). The envelope glycoprotein is encoded by VSV-G (vesicular stomatitis virus) and is expressed under a strong promoter, such as CMV. The *rev* gene is split from the packaging plasmid and is provided on a separate plasmid construct. The packaging plasmid harbors the viral *gag* and *pol* genes, and notably lacks the *tat* regulatory gene

Lentiviral vector systems that are derived from the HIV-1 virus have evolved through the years. These advancements have been made in part to mitigate the potential risks associated with the virus that the platform is based on. One obvious safety consideration in designing a lentiviral vector system for gene therapy is the unintended generation of replication-competent provirus. The first generation of HIV-1-based vectors retained most of the viral genome within the *trans* packaging construct, including the viral core, regulatory protein coding sequences, and accessory regulatory genes.^256^ In the three-plasmid design to generate vector, the *env* gene is replaced by the glycoprotein of vesicular stomatitis virus (VSV-G) and is separately provided in *trans* by a second plasmid. In this way, pseudotyping the viral particle with VSV-G facilitates viral entry independent of its native host cell receptor. The therapeutic transgene is provided in a third plasmid construct along with the *cis*-acting elements that confer viral encapsidation. The second generation of safer lentiviral vectors are devoid of *vif*, *vpr*, *vpu*, and *nef* auxiliary genes, which promote viral proliferation and infection.^267^ To limit the formation of unintended replication-competent provirus, third-generation vectors that lack the *tat* and *rev* regulatory genes in the packaging construct were developed (Fig. 5).^268^ The *rev* gene, which is required for replication, is supplied in *trans*, creating a conditional packaging platform. The safety profile of the third-generation lentiviral system is further improved by deleting part of the 3′-LTR, which contains the TATA box and transcription factor-binding sites; thus, creating a vector packaging system that is self-inactivating.^269^

Additional modifications have been made to improve the expression and transduction efficiency of lentiviral vectors. Incorporating transcriptional regulatory elements, such as a central polypurine tract (*cppt*) and a matrix attachment region (*MAR*) in the *cis* expression vector augments viral transduction.^270^ Inclusion of scaffold attachment regions from the β-interferon gene into the vector design enhances the expression of vector transgenes in quiescent T cells.^271^ In addition, incorporating woodchuck hepatitis virus post-transcriptional regulatory element (WPRE) as a posttranscriptional regulatory element in the 3′-untranslated region of the ORF significantly enhances transgene expression.^272^

Similar to other viral vectors, achieving tissue or cell-type specificity is a key component for designing efficient and safe lentiviral vectors. Numerous tissues or cell types have been specifically targeted by employing tissue-specific promoters to drive transgene expression.^273,274^ For instance, temporal regulation of lentiviral vector expression can be achieved by using tetracycline-inducible promoters.^275^ In addition, cell-specific targeting can be improved by utilizing endogenous miRNAs to posttranscriptionally regulate transgene expression, thereby reducing immune response mounted against the transgene.^156^ Pseudotyping has paramount importance to the success of transducing the desired cell or tissue type. For example, lentivirus pseudotyped with glycoproteins of simian immunodeficiency virus and Ebola virus have improved transduction to the retina and hematopoietic stem cells (HSCs), respectively.^276,277^

#### Production platforms

Large-scale good manufacturing practice (GMP) of lentiviral vectors for clinical application involves intricate production, purification, and quality assessment steps. The process of lentiviral vector production can be roughly divided into upstream production and downstream purification procedures. For clinical applications, viral production of third-generation vectors can be achieved by quadruple plasmid transfection with the transfer vector, along with the three separate plasmids carrying packaging and helper genes.^278–280^ HEK293 or HEK293T producer cell lines are routinely used. Although the HEK293T cell line is preferred, as it confers higher viral titers,^278,281^ the HEK293T line carries the oncogenic simian vacuolating virus 40 large T antigen, which poses safety concerns. Although the four-plasmid transfection method is highly modular, batch-to-batch variability in viral titers remains a challenge.

Alternatively, lentivirus vectors can be manufactured by using a stable producer cell line that harbors functional lentiviral helper and packaging genes. In such stable producer cell lines, tetracycline-inducible systems are customarily used to control VSV-G and *gag-pol* expression, which would otherwise be toxic to cells.^282,283^ Pseudotyping with VSV-G also enhances the stability of the viral particle during vector manufacturing.^284,285^ Cumate- and tetracycline-inducible systems have been concomitantly used to limit leaky viral protein expression.^286,287^ In addition to inducible stable cell lines, constitutive packaging cell lines have been developed for a number of envelope proteins that are less toxic than VSV-G, such as cocal^288^ and RD114-TR.^289^ The use of producer cell lines has an edge over transient transfection methods in terms of cost, reproducibility, and scalability. However, it can be cumbersome to produce a stable cell line for each pseudotyping envelope protein and helper gene combination.

Suspension cell culture production is becoming a widely adopted method for scalable large-scale lentiviral manufacturing because of its superiority over adherent cell culture production schemes. Different cell clones amenable for suspension culture system have been developed.^287,290^ Serum-free media is commonly used for GMP-manufacturing of large-scale vector intended for clinical use, as this reduces biological contaminants inherent to sera.^291,292^ In downstream processes, initial centrifugation and filtration steps help to remove cellular debris. These procedures are followed by purification of viral particles with various chromatographic techniques. Buffer exchange and viral particle concentration can be carried out by ultracentrifugation and tangential flow filtration.^293–295^ The purification process needs to be optimized for each pseudotyping strategy. Large-scale production of lentiviral vector for clinical application needs to be in compliance with current GMPs (cGMPs), which include the use of cGMP-certified reagents and manipulation under aseptic conditions.

#### Therapeutic lentiviral vectors and commercial availability

The HIV virus has killed about 32 million people to date (www.who.int/gho/hiv/en/). In transforming this lethal pathogenic virus into a delivery vehicle for gene therapy, we have come a long way towards understanding lentiviruses. For instance, in recent years, ex vivo applications for lentiviruses in the form of generating chimeric antigen receptor (CAR) T cells for cancer immunotherapy have revolutionized how cancer is treated.

Engineered CAR-T cells have been developed to specifically target cancer-related antigens. The use of lentiviral vectors is critical for developing CAR-T cells to treat refractory hematologic malignancies. This approach has been demonstrated in a safety assessment clinical trial that involved 30 children and adult patients with relapsed and refractory acute lymphoblastic leukemia (ALL).^296^ In these patients, autologous T cells were transduced with lentiviral vectors carrying a transgene to express a CAR that binds CD19. The study showed that a remission rate as high as 90% could be achieved in patients who underwent the CAR-T cell infusion treatment.^296^ Going by the commercial name Kymriah, the treatment later became the first drug to be approved by the FDA to treat pediatric B-cell ALL. The second CAR-T cell-based drug approved by the FDA for the treatment of refractory large B cell lymphoma, named Yescarta, uses gammaretrovirus to stably deliver the CAR gene construct to cells.^297^

#### Lentiviral vectors in clinical trials

Lentiviral vectors have been tested in many successful clinical trials. The first endeavor employed a lentiviral vector to treat HIV-positive patients via ex vivo transduction of patients’ CD4 cells by antisense sequence against the wild-type HIV viral envelope gene.^298^ The study demonstrated that the vector lowered viral load and did not result in any major adverse effects. To date, there are more than a dozen completed clinical trials that have used lentiviral vectors for treating a range of diseases, including metabolic disorders, cancers, immune disorders, and rare congenital diseases (Table 3). Moreover, due to its safety profile compared to other retroviruses, the number of ongoing trials that use lentiviral vector is dramatically increasing.

**Table 3:** A selection of ongoing and completed clinical trials employing lentiviral vector

Condition	Intervention	Sponsor/collaborators	Trial stage	NCT number
Acute lymphoblastic leukemia	CART-19 T cells	Union Stem Cell & Gene Engineering Co. Ltd, Juventas Cell Therapy Ltd	Phase I	NCT02975687
		University of Pennsylvania		NCT01029366
		University of Pennsylvania	Phase II	NCT02030847
ADA-SCID	Autologous EFS-ADA LV CD34+ cells	Orchard Therapeutics, NIAID, NHGRI, NHLBI, UCLA	Phase I/II	NCT01852071
		Orchard Therapeutics, UCLA	Phase III	NCT04140539
		NHGRI, UCLA, Duke University, NCI, CC	Phase I	NCT02022696
		Orchard Therapeutics, CIRM, UCLA	Phase I/II	NCT02999984
		NHS Foundation Trust, Orchard Therapeutics	Phase I/II	NCT01380990
Age-related macular degeneration	Endostatin and Angiostatin LV	Oxford BioMedica	Phase I	NCT01301443
Non-Hodgkin lymphoma	CART-19 T cells	Peking Union Medical College Hospital, Cellular Biomedicine Group Ltd	Phase I	NCT03483688
Cerebral adrenoleukodystrophy (CALD)	Autologous ABCD1 LV CD34+ cells	Bluebird Bio	Phase II/III	NCT01896102
β-Thalassemia	Autologous βA-T87Q-globin LV CD34+ cells	Bluebird Bio	Phase I/II	NCT01745120
		Bluebird Bio	Phase III	NCT03207009 NCT02906202
β-Thalassemia major, sickle cell disease	Autologous βA-T87Q-globin LV CD34+ cell	Bluebird Bio	Phase I/II	NCT02151526
Sickle cell disease	Autologous βA-T87Q-globin LV CD34+ cells	Bluebird Bio	Phase III	NCT04293185
Fanconi anemia	Autologous FANCA LV CD34+ cells	Hospital Universitari Vall d’Hebron Research Institute, CIEMAT, CIBERER	Phase II	NCT02931071
HIV infection	Autologous C34-CXCR4 LV CD4 T cells	University of Pennsylvania	Early Phase I	NCT03020524
	Wt gag-TCR and α/6-gag-TCR-modified T cells	University of Pennsylvania, Adaptimmune	Phase I	NCT00991224
	HIV antigen LV	Theravectys S.A.	Phase I/II	NCT02054286
Lymphoma	rHIV7-shI-TAR-CCR5RZ LV HPCs	City of Hope Medical Center, NCI	Phase I	NCT00569985
Multiple myeloma	CART-19 T cells	University of Pennsylvania	Phase I	NCT02135406
Refractory diffuse large B-cell lymphoma	CART-19 T cells	Cellular Biomedicine Group Ltd, Nanjing Medical University	Phase I	NCT02976857
Covid-19 infection	SARS-CoV-2 antigen in aAPC	Shenzhen Geno-Immune Medical Institute	Phase I	NCT04299724
Wiskott–Aldrich syndrome	Autologous WAS LV CD34+ cells	Genethon, Hôpital Necker-Enfants Malades	Phase I/II	NCT01347346
		Genethon, NHS Foundation Trust, Institute of Child Health	Phase I/II	NCT01347242

*ADA-SCID* adenosine deaminase severe combined immunodeficiency, *CART-19 T* chimeric antigen receptor 19 T cells, *CC* National Institutes of Health Clinical Center, *EFS-ADA* elongation factor 1α promoter driving adenosine deaminase, *FANCA* Fanconi anemia complementation group gene, *HIV* human immunodeficiency viruses, *HPCs* hematopoietic progenitor cells, *LV* lentiviral vector, *NCI* National Cancer Institute, *NHGRI* National Human Genome Research Institute, *NHLBI* National Heart, Lung, and Blood Institute, *NIAID* National Institute of Allergy and Infectious Diseases, *SARS-CoV-2* severe acute respiratory syndrome coronavirus, *TCR* T-cell receptor, *UCLA* University of California Los Angeles, *WAS* Wiskott–Aldrich syndrome gene, *wt* wild type.

In particular, the use of lentiviruses in ex vivo gene therapy strategies for treating genetic diseases have enjoyed substantial advancements. For example, 18 β-thalassemia patients carrying mutations in the HBB globin gene received infusion of autologous CD34+ cells transduced with lentiviral vectors encoding the human βA-T87Q-globin gene in phase I/II studies.^299^ The treatment proved to be successful in replacing long-term allogeneic hematopoietic cell transplantations. In patient follow-ups, there were no significant toxicity effects related to the vector after three years post infusion. This effort has led to the development of LentiGlobinBB305, which has now advanced to a multinational phase III clinical trial for the treatment of β-thalassemia (NCT03207009 and NCT02906202) and sickle cell disease (NCT04293185). Similarly, a lentiviral based gene therapy approach has shown promising results for the treatment of cerebral adrenoleukodystrophy, which typically has a very poor prognosis without allogeneic hematopoietic stem cell transplantation (HSCT). Nearly 90% of cerebral adrenoleukodystrophy patients who received hematopoietic stem cells transduced with lentiviral vectors carrying the *ABCD1* transgene have overcome major functional impairment.^300^ No genotoxicity or adverse effects related to the treatment have been reported for any of the test subjects.

Ex vivo gene therapy has also been a viable alternative to allogeneic HSCT treatment of inherited primary immune deficiency disorders. To this end, lentiviral gene therapy has been demonstrated to be safe in patients with ADA-SCID that is caused by a mutation in the *ADA* gene. In a non-randomized clinical trial conducted at two different sites in the USA, 30 pediatric ADA-SCID patients were infused with autologous CD34+ hematopoietic stem/progenitor cells transduced with lentiviral vectors containing the *ADA* transgene.^301^ With the exception of one patient exhibiting non-engraftment, all other patients were taken off of enzyme replacement therapy and showed event-free survival beyond two years post treatment. Following this success, the lentiviral vector drug named OTL-101, entered a phase III clinical trial for the treatment of ADA-SCID (NCT04140539). In another primary immune deficiency disorder, eight pediatric patients with Wiskott–Aldrich syndrome were infused with autologous CD34+ transduced with a lentiviral vector carrying the *WAS* transgene. Interim analysis at 3.6 years post treatment showed an overall 100% survival rate with improved immunity and removal from immunoglobulin supplementation for seven of the eight treated patients.^302^

In comparison to gammaretroviral vectors, lentiviral vectors are preferentially used in many advanced (phase III) clinical trials for CAR-T-cell therapies. Ongoing phase III clinical trials for CAR-T cells targeting refractory B-cell lymphoma (NCT03391726), ALL (NCT03027739 and NCT03937544), and refractory B-cell acute myeloid leukemia (NCT03631576) were developed by lentiviral vectors.

Engineering of T-cell receptors (TCRs) to specifically recognize cancer-specific proteins is another cancer immunotherapy approach being explored. In a preclinical study, lentiviral vectors were successfully used to transduce hematopoietic stem cells with NY-ESO-1 TCR, with no measurable genotoxicity.^303^ A recently reported clinical trial indicated that a TCR targeting the NY-ESO-1 antigen in multiple myeloma patients had good efficacy and minimal toxicity (NCT01352286).^304^

Lentiviral vectors are also being tested to tackle viral infection including the development of novel vaccines against COVID-19. In two recently launched clinicals, lentiviral vectors are being used to express synthetic viral minigenes and immune modulatory genes to engineer artificial APCs to activate the immune system against COVID-19 (NCT04299724).

#### Challenges

An early clinical trial that used a gammaretroviral vector to treat X-linked SCID led to serious adverse effects.^305^ Four out of the nine patients who received the treatment went on to develop T-cell leukemias, as a result of insertional mutagenesis. Thus, lentiviral vectors have become the preferred vehicle for transgene delivery, owing to their reduced genotoxicity profile when compared to gammaretroviral vectors.^306–308^ Although both vectors preferentially integrate into transcriptionally active regions, one possible factor contributing to differences in genotoxicity is that gammaretroviral vectors often integrate within the vicinity of transcriptional start sites, and has affinities toward oncogenes.^307,309–311^ The use of third-generation, self-inactivating lentiviral vectors have helped to mitigate the risk of insertional mutagenesis.^269^ Nonetheless, it has been reported that even self-inactivating lentiviral vectors with strong promoter and enhancer elements can activate neighboring genes. Incorporation of insulator sequences to neutralize the effect of enhancers acting in trans can reduce these effects.^312–315^ In addition, integration can potentially form chimeric gene fusions made up of proviral and host sequences.^316–318^ Finally, lentiviral vectors have been shown to cause aberrant splicing of cellular transcripts.^318,319^

#### The future for lentivirus vectors

The number of viral vectors being developed as delivery platforms for genetic vaccines is increasing. Due to their ability to transduce non-dividing cells, such as dendritic cells, recombinant lentiviral vectors have been shown to elicit B cell- and T cell-mediated immunity against infectious diseases and different tumors.^320^ Recent advances in the development of non-integrating lentiviral vectors have greatly reduced insertional mutagenesis.^321^ Another advantage of non-integrating lentiviral vectors is their transient expression in actively dividing cells, where sustained transgene expression is not necessary. Application of non-integrating lentiviral vectors to circumvent immune responses mounted against prolonged expression of genome editing tools, such as CRISPR/Cas, facilitates the use of such systems in therapeutic gene editing.^322^ In addition to non-integrating lentiviruses, self-restricted CRISPR systems with shortened duration of *cas9* expression, could reduce off-target effects and enhance CRISPR-based therapeutics.^323^

Integrase-defective lentiviral vectors have been designed as a vaccine platform to deliver vaccination against influenza and malaria antigens.^324,325^ Such lentiviral vectors are being continuously developed and optimized, and could enjoy a broader application in future gene therapies. In addition, the development of safer vectors via photo-switchable non-canonical amino acids to regulate transgene expression in a spatial and temporal manner,^326^ also represent the next generation of lentiviral vectors.

Lentiviral vector has become one of the preferred tools for ex vivo transgene delivery for gene therapy, because it has many attractive features. These vectors have proven to be invaluable for ex vivo gene correction and gene transfer. Lentiviral vector systems have evolved a great deal and are increasingly being improved. This effort will result in wider adoption of lentiviral vectors to treat human diseases.

## Closing remarks

In this review, we have discussed the current state of viral vectors, specifically those based on Ad, AAV, and lentivirus. It is abundantly clear that the future for viral-based vectors is bright and the ability to address many human genetic diseases is within reach. Unfortunately, the current tools at our disposal are still wrought with complications, and for many diseases, a long path awaits until viable treatments are available. Further exploration into viral biology, as well as advanced and interdisciplinary approaches are needed to overcome the current challenges that still curb the promise for the numerous vector platforms currently in preclinical and clinical testing phases. What the past 30 years has taught us is that every great idea has unexpected pitfalls. Nevertheless, every obstacle that is faced and overcome gifts us with a clearer sense of where our goals towards improving human health are set.
